# Bioceramic Scaffolds with Antioxidative Functions for ROS Scavenging and Osteochondral Regeneration

**DOI:** 10.1002/advs.202105727

**Published:** 2022-02-19

**Authors:** Cuijun Deng, Quan Zhou, Meng Zhang, Tian Li, Haotian Chen, Chang Xu, Qishuai Feng, Xin Wang, Feng Yin, Yu Cheng, Chengtie Wu

**Affiliations:** ^1^ Translational Medical Center for Stem Cell Therapy & Institute for Regenerative Medicine Shanghai East Hospital Tongji University School of Medicine 1800 Yuntai Road Shanghai 200123 P.R. China; ^2^ State Key Laboratory of High Performance Ceramics and Superfine Microstructure Shanghai Institute of Ceramics Chinese Academy of Sciences Shanghai 200050 P.R. China; ^3^ Department of Joint Surgery Shanghai East Hospital School of Medicine Tongji University Shanghai 200123 P.R. China

**Keywords:** antioxidant defense, arthritis therapy, bioceramic scaffolds, osteochondral regeneration, ROS scavenging

## Abstract

Osteoarthritis (OA) is a degenerative disease that involves excess reactive oxygen species (ROS) and osteochondral defects. Although multiple approaches have been developed for osteochondral regeneration, how to balance the biochemical and physical microenvironment in OA remains a big challenge. In this study, a bioceramic scaffold by 3D printed akermanite (AKT) integrated with hair‐derived antioxidative nanoparticles (HNPs)/microparticles (HMPs) for ROS scavenging and osteochondral regeneration has been developed. The prepared bioscaffold with multi‐mimetic enzyme effects, which can scavenge a broad spectrum of free radicals in OA, can protect chondrocytes under the ROS microenvironment. Importantly, the bioscaffold can distinctly stimulate the proliferation and maturation of chondrocytes due to the stimulation of the glucose transporter pathway (GLUT) via HNPs/HMPs. Furthermore, it significantly accelerated osteogenic differentiation of bone marrow mesenchymal stem cells (BMSCs). In vivo results showed that the bioscaffold can effectively enhance the osteochondral regeneration compared to the unmodified scaffold. The work shows that integration of antioxidant and mechanical properties via the bioscaffold is a promising strategy for osteochondral regeneration in OA treatment.

## Graphical abstract

A bioceramic scaffold by 3D printed akermanite (AKT) integrated with hair‐derived antioxidative nanoparticles (HNPs)/microparticles (HMPs) for ROS scavenging and osteochondral regeneration has been developed. The bioscaffold with multi‐mimetic enzyme effects can stimulate osteogenic differentiation of rBMSCs and promoted chondrocytes maturation, which further protects chondrocytes from OA.

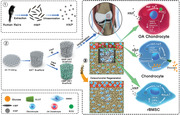

## 1 Introduction

Osteoarthritis (OA), a chronic degenerative disease, which is characterized by chronic inflammation and cartilage degradation, leading to cartilage lesion and subchondral bone defect in terminal OA.^[^ ^1^, ^2^ ^]^ Current treatment strategies include microfracture, autograft/allograft, and joint replacement. Although microfracture can partly repair cartilage, the newly formed tissue is inferior and consisted of fibrocartilage.^[^ ^3^ ^]^ Autograft/allograft might lead to secondary trauma, pain, or immune rejection, which limits their applications in osteochondral reconstruction.^[^ ^4^, ^5^ ^]^ Arthroplasty and joint replacement are regarded as feasible therapeutic strategies for terminal OA but there is a lifespan limit for the artificial implants.

Tissue engineering scaffolds are alternative strategies to overcome the current treatment limitations in order to permanently repair the damaged tissue and recover osteochondral defects.^[^ ^6^, ^7^ ^]^ A growing number of bioscaffolds mimicking the composition and physical properties of natural bones have been designed to treat OA. For instance, 3D printing bioceramic scaffolds offer good physical properties and biocompatibility for osteochondral regeneration.^[^ ^8^, ^9^, ^10^ ^]^ Ion‐doped bioceramic scaffolds and micro/nanometer‐structured scaffolds can provide the mechanical support and release ions simultaneously to facilitate cartilage and subchondral bone regeneration.^[^ ^11^, ^12^ ^]^ However, these bioscaffold‐based strategies often suffer from insufficient regeneration of osteochondral tissues hindered by the strong inflammatory environment of OA. Therefore, an ideal bioscaffold should preserve the capability to balance both physical and biochemical microenvironments in OA.

In OA development and progression, multiple ROS and reactive nitrogen species (RNS) are generated due to oxidative stress in the inflammatory environment.^[^ ^13^, ^14^, ^15^ ^]^ High levels of ROS inhibited matrix synthesis and impairment of the natural osteochondral regeneration process by damaging the mitochondrial DNA and inducing chondrocyte apoptosis.^[^ ^16^, ^17^ ^]^ Moreover, ROS leads to cartilage degeneration and calcification by inhibiting extracellular matrix (ECM) formation and facilitating ECM catabolism.^[^ ^18^ ^]^ Therefore, it is essential to suppress ROS during the osteochondral regeneration process. Although antioxidants, such as N‐acetylcysteine and melatonin, have been tested, they show fast metabolism in the knuckle.^[^ ^19^, ^20^ ^]^ Furthermore, metal nanomaterials and nanozymes have been developed to balance the oxidative environment for OA treatment.^[^ ^20^, ^21^, ^22^ ^]^ However, lacking of mechanical properties limits their further applications for osteochondral regeneration.^[^ ^23^ ^]^ Hence, developing an antioxidative tissue engineering scaffold that could simultaneously regulate the biochemical and physical microenvironment in OA is urgently needed.

Previously, it was reported that the nanoparticles and microparticles from human hair mainly consist of melanin and keratin, which are ideal biomedical materials for tissue engineering.^[^ ^24^ ^]^ Keratin‐based biomaterials play superior performance in tissue regeneration.^[^ ^25^ ^]^ Recent studies demonstrated that melanin possesses excellent activities in scavenging oxidant stress and protecting healthy tissue.^[^ ^26^, ^27^, ^28^ ^]^ The aforementioned studies indicate that HNPs and HMPs hold great potential for ROS scavenging and osteochondral regeneration. Furthermore, AKT is a typical bioceramic with distinct bone‐forming bioactivity.^[^ ^29^, ^30^ ^]^ Hence, we developed a unique bioceramic scaffold by 3D printed AKT integrated with HNPs/ HMPs for osteochondral regeneration in OA. HNPs and HMPs with excellent biocompatibility and biosafety were isolated from human hair and showed good ROS scavenging activity. Their chondrogenic and osteogenesis induction effects were discovered. To prepare a stable bioceramic scaffold with antioxidative activity, HNPs/HMPs were further integrated with AKT scaffolds via a gentle apatite mineralization method to achieve both antioxidative and mechanical properties. We hypothesized that HNPs/HMPs integrated with AKT scaffold (HNP/HMP‐AKT) might synergistically balance the biochemical and physical microenvironment in OA, which could promote osteochondral regeneration. The underlying mechanism of the bioscaffolds for maturation of chondrocytes, osteogenic differentiation of BMSCs, and in vivo osteochondral regeneration efficacy was investigated.

## 2 Results

### 2.1 HNPs and HMPs Showed the Multi‐Mimetic Enzyme Effects to Scavenge Free Radicals

**Figure 1:**
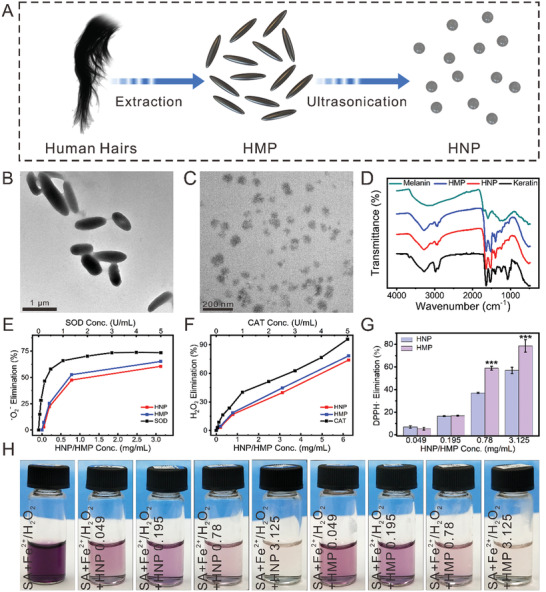
Preparation and characterization of HNPs and HMPs. A) Preparation of HMP and HNP. B) and C) showed the TEM images of HMPs and HNPs, respectively. D) The FT‐IR spectrum of HNPs and HMPs. Compared with artificially synthesized melanin, HNPs and HMPs had a distinct absorption peak similar to keratin (3000–2800 cm^−1^), which reflected the C–H absorption of the alkane structure and demonstrating the existence of great amount of keratin. E) SOD‐like activity of HNPs and HMPs. F) CAT‐like activity of HNPs and HMPs. G) Nitrogen free radical scavenging effect of HNPs and HMPs. As compared to the same concentration of HNPs, HMPs significantly scavenged DPPH free radicals within the concentration range of 0.78–3.125 mg ml^−1^. Repeat number: *n* = 5. ***p < 0.001 (one tailed Student *t*‐test). Error bars represent mean ± SD. H) Hydroxyl radical scavenging effect of HNPs and HMPs. SA reacted with H_2_O_2_ or Fe^2+^ were used as controls. HNPs and HMPs have CAT‐like and SOD‐like activities, and possessed good antioxidant effects for nitrogen free radical and hydroxyl radical scavenging.

Schematic illustration of HNPs and HMPs preparation was shown in **Figure** **1**A. In this study, HMPs were isolated from the black human hair through an alkaline dissolving method.^[^ ^24^ ^]^ TEM images showed that HMPs had a rod‐like morphology with an average length of approximately 1.0 µm and an average width of 350 ± 50 nm (Figure 1B). HMPs were decomposed into HNPs after ultrasonic treatment, and the average diameter of the HNPs was 60 ± 15 nm (Figure 1C). The size of both HNPs and HMPs was similar in different pools (Figure S1A_1–5_, Figure SB_1–5_, Supporting Information). Furthermore, dynamic light scattering (DLS) analysis was utilized to confirm the particle size of HNPs and HMPs (Figure S1C, Supporting Information). As reported previously, human hair mainly consists of keratins and melanins.^[^ ^24^ ^]^ To further confirm the compositions of HNPs and HMPs, FT‐IR and UV‐vis analyses were conducted. The results indicated that HNPs and HMPs had a distinct absorption peak similar to that of keratin (3000–2800 cm^−1^) and possessed a rapidly increased absorbance value in the UV region, which demonstrated that keratins and melanins were the main components of HNPs and HMPs (Figure 1D, Figure S1D, Supporting Information).

Furthermore, the antioxidant activity of HNPs and HMPs was evaluated by measuring the free radicals and H_2_O_2_ scavenging capacity. Superoxide anion is one type of free radical that is harmful to cartilage and bone.^[^ ^31^ ^]^ Superoxide dismutase (SOD) is an antioxidant enzyme in organisms, that can catalyze superoxide anion disproportionation to generate oxygen and hydrogen peroxides. To investigate the SOD‐like activity of HNPs and HMPs, a SOD detection kit was used. HNPs and HMPs showed a similar trend to that of the natural SOD enzyme in scavenging superoxide anions, which indicated that HNPs and HMPs had a decent SOD‐like activity (Figure 1E, Figure S1E, Supporting Information). To further evaluate whether HNP and HMP could scavenge the hydrogen peroxides generated by SOD disproportionation, the catalase (CAT)‐like activity was measured. A CAT assay kit and a portable dissolved oxygen tester were used to study the CAT‐like activity of HNPs and HMPs. The results indicated that HNPs and HMPs had a similar role as that of CAT in H_2_O_2_ clearance, and the oxygen production increased with the enhancement of HNPs or HMPs (Figure 1F, Figure S1F, Supporting Information). To further investigate the nitrogen free radical scavenging effect, DPPH free radicals were employed. DPPH free radicals are a typical nitrogen free radical used for evaluating the antioxidation capability of an antioxidant, which has dramatic absorbance at 400–600 nm wavelengths. The optical images and UV‐vis spectra indicated that the absorbance value decreased dramatically with the increased concentrations of HNPs and HMPs, and the clearance efficiency increased to 80% after reacting with HMPs (3.125 mg ml^−1^) for 30 min (Figure 1G and Figure S1H,I, Supporting Information). In addition to nitrogen free radical scavenging effect, hydroxyl radical scavenging activity was investigated. The Fenton reaction was used to generate hydroxyl radicals. After treatment with HNPs or HMPs, the residual hydroxyl radicals were reacted with salicylic acid (SA) to generate the characteristic absorbance at 510 nm. The UV‐vis analysis and digital photos demonstrated that HNPs and HMPs significantly scavenged hydroxyl radicals within a concentration range of 0.049–3.125 mg ml^−1^ (Figure 1H). Specifically, solutions reacted with 3.125 mg ml^−1^ of HNPs or HMPs were almost colorless, and the UV‐vis spectrum showed that up to 95% clearance efficiency of hydroxyl radicals could be achieved (Figure S1G, Supporting Information).

### 2.2 HNP and HMP Endowed Bioceramic Scaffolds with Antioxidant Activities

**Figure 2:**
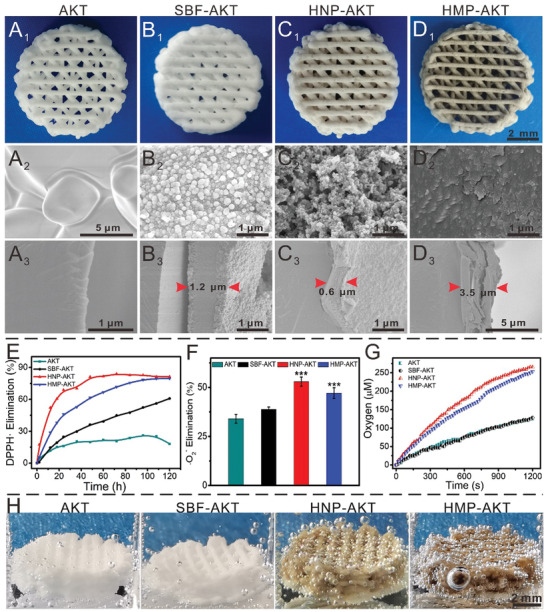
Characterization of AKT scaffolds before and after modifying with HNPs/HMPs. Photographs of A_1_) pure AKT scaffold, B_1_) SBF‐AKT scaffold, C_1_) HNP‐AKT scaffold, D_1_) HMP‐AKT scaffold. SEM images of the surface of A_2_) pure AKT scaffold, B_2_) SBF‐AKT scaffold, C_2_) HNP‐AKT scaffold, D_2_) HMP‐AKT scaffold. A_3_–D_3_) The cross‐section surface of scaffolds. E) Nitrogen free radical scavenging effects in 120 h. F) Superoxide anion radical scavenging effects. As compared with AKT scaffolds, HNP‐AKT scaffolds and HMP‐AKT scaffolds distinctly scavenged superoxide anion radicals. Repeat number: *n* = 5. ***p < 0.001 (one‐way ANOVA followed by Dunnett's multiple comparisons test). Error bars represent mean ± SD. G) The oxygen is produced in 25 mM of H_2_O_2_. H) Photographs of the oxygen bubbles in scaffolds after treating with H_2_O_2_. The surface of pure AKT scaffold displayed a dense morphology, while the HMP‐AKT scaffold has a rough surface, and the SBF‐AKT scaffold and HNP‐AKT scaffold were covered with different sizes of nanoparticles. The coating thickness of SBF‐AKT, HNP‐AKT, and HMP‐AKT were about 1.2 µm, 0.6 µm, and 3.5 µm, respectively. Furthermore, HNP‐AKT and HMP‐AKT scaffolds showed the ability to significantly scavenged nitrogen free radical, superoxide anion radical, and H_2_O_2_, as well as have CAT like activity.

In this study, the well‐designed morphology of pure AKT scaffolds was successfully developed via a 3D printing technology. Then, HNPs/HMPs were integrated into AKT scaffolds via an apatite mineralization method.^[^ ^32^ ^]^ SBF was used as a biomineralized solution for dispersing HNPs and HMPs. The scaffolds obtained from treating with SBF+HNPs/HMPs were HNP‐AKT/HMP‐AKT scaffolds, while the scaffolds treated with SBF alone were SBF‐AKT scaffolds. In digital photos, pure AKT scaffolds displayed a uniform macropore morphology with a well‐crystallized surface (**Figure** **2**A_1,2_). After coating scaffolds with HNPs/HMPs, they maintained the well‐designed microporous structure (Figure 2C_1_, Figure 2D_1_). Under SEM, the pure AKT scaffold showed a dense and well‐crystallized surface (Figure 2A_2,3_). However, the HMP‐AKT scaffold had a rough surface, whereas the SBF‐AKT scaffold and HNP‐AKT scaffold were covered with different sizes of nanoparticles (Figure 2B_2_–D_2_). Using different biomineralized raw materials, different surface structures and coating thicknesses were obtained (Figure 2B_3_–D_3_). The scaffolds only treated with SBF had a globular nanoflower surface with a thickness of 1.2 µm. When combined with HNPs, a dense nanoparticle surface approximately 0.6 µm of thick was obtained in HNP‐AKT scaffolds. Instead of HNPs, HMPs were dispersed in SBF and then used to incubate with AKT scaffolds. The obtained HMP‐AKT scaffolds had a uniform rough surface with a thickness of 3.5 µm. Based on the previous studies, surface modification may change the compressive strength of scaffolds. To investigate mechanical properties, the scaffolds were prepared with a height of 10.0 ± 0.705 mm and a diameter of 8.0 ± 0.685 mm (Figure S2A, Supporting Information). The results indicated that the compressive strength of AKT scaffolds had no significant difference before and after coating with HNPs or HMPs, which was approximately 10 MPa (Figure S2B, Supporting Information). After incubating at 37 °C in Tris–HCl solution for 5 weeks, the degradation of scaffolds had no distinct difference in each group, and the weight loss of scaffolds was approximately 15% (Figure S2C, Supporting Information). Furthermore, the release profile of Si, Ca, and Mg in Tris–HCl solution was investigated. Si, Ca, and Mg release increased over time and the release rates had no obvious difference among groups (Figure S2D–F, Supporting Information). In the optical images, color changes in scaffolds indicated that HNPs and HMPs were successfully coated on AKT scaffolds (Figure S2A, Supporting Information). Compared to the FT‐IR spectra of pure HNPs and HMPs, the FT‐IR spectra of the HNP‐AKT and HMP‐AKT scaffolds further confirmed the existence of HNPs and HMPs in the scaffolds (Figure S2G, Supporting Information). Additionally, HNPs and HMPs could withstand release from scaffolds during the incubation period (Figure S2H, Supporting Information).

Based on the superior antioxidant activity of HNPs and HMPs, the antioxidant activity of HNP‐AKT and HMP‐AKT scaffolds was systematically studied. DPPH free radical scavenging activity was previously studied. In Figure S3A_1–4_ (Supporting Information), the color of the DPPH free radical solution significantly faded as time increased in the HNP‐AKT and HMP‐AKT groups, and that of the pure DPPH and SBF‐AKT groups was slightly faded, whereas the AKT group changed to red‐orange color. UV‐vis spectra further confirmed that HNP‐AKT and HMP‐AKT scaffolds obviously scavenged DPPH free radicals as compared to the scavenging ability of AKT scaffolds and SBF‐AKT scaffolds (Figure S3B_1–4_, Supporting Information). The cumulative clearance of DPPH in the HNP‐AKT and HMP‐AKT groups was approximately 90% at 120 h (Figure 2E). Furthermore, the superoxide anion scavenging effect of scaffolds was assessed by using a SOD enzyme assay kit. It was found HNP‐AKT scaffolds significantly scavenged superoxide anions as compared with pure AKT scaffolds (Figure 2F). Further studies demonstrated that HNP‐AKT and HMP‐AKT scaffolds had CAT‐like activity, which could react with H_2_O_2_ to generate oxygen and water (Figure 2G, Figure S2I, Supporting Information). Oxygen bubbles generated in scaffolds further confirmed that HNP‐AKT and HMP‐AKT scaffolds possessed the CAT‐like activity (Figure 2H).

### 2.3 HNPs and HMPs Promoted Cell Adhesion and Proliferation in Scaffolds

**Figure 3:**
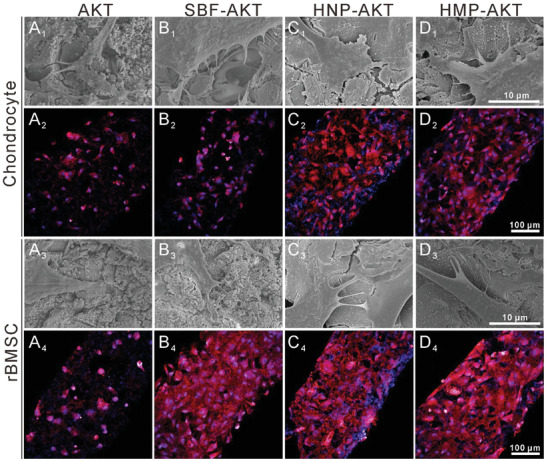
Morphology of chondrocytes and rBMSCs cultured on AKT scaffolds before and after biofunctionalization. A_1_–D_1_) and A_3_–D_3_) showed the SEM images of chondrocytes and rBMSCs cultured on the surface of scaffolds, respectively. A_2_–D_2_) and A_4_–D_4_) displayed the CLSM images of chondrocytes and rBMSCs incubated in the scaffolds, respectively.

A cell counting kit‐8 (CCK‐8) assay was used to evaluate the adhesion and proliferation of chondrocytes and rBMSCs in scaffolds. After 24 h of incubation, HNP‐AKT scaffolds significantly promoted the adhesion of chondrocytes and rBMSCs as compared with the adhesion observed with SBF‐AKT scaffolds (Figure S4A,B, Supporting Information). The cell attachment rate of chondrocytes in HMP‐AKT scaffolds was distinctly higher than that of SBF‐AKT scaffolds at 24 h, whereas the rBMSCs attached to HMP‐AKT scaffolds were similar with the SBF‐AKT scaffold, and yet both were higher than that of AKT scaffolds. Furthermore, the proliferation of rBMSCs in HNP‐AKT and HMP‐AKT scaffolds was obviously superior to that of SBF‐AKT and AKT scaffolds, whereas the proliferation activity of chondrocytes was similar in the experimental groups (Figure S4C,D, Supporting Information). SEM and CLSM were further utilized to evaluate cell attachment and distribution in scaffolds. CLSM analysis further confirmed the stimulating effect of HNP‐AKT and HMP‐AKT scaffolds on rBMSCs and chondrocytes. In CLSM images, the chondrocytes and rBMSCs in the HNP‐AKT and HMP‐AKT groups showed more widespread cytoskeletons than those in the AKT group, whereas the cells in the AKT scaffolds showed hardly any cytoskeleton. Moreover, there were more cells attached to the HNP‐AKT and HMP‐AKT scaffolds than to the AKT scaffolds (**Figure** **3**A_2_–D_2_, 3A_4_–D_4__,_ and Figure S5, Supporting Information). SEM images further showed that chondrocytes and rBMSCs were widespread well in the HNP‐AKT and HMP‐AKT groups, and the cells showed better‐defined cytoskeletons and plump morphology as compared to those of the SBF‐AKT and AKT groups (Figure 3A_1_–D_1_, 3A_3_–D_3,_ and Figure S6, Supporting Information).

### 2.4 HNPs and HMPs Protected Chondrocytes from OA Environment

**Figure 4:**
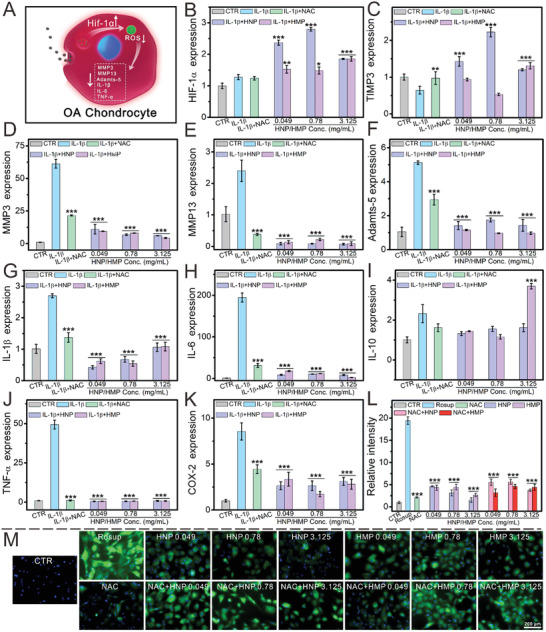
HNPs and HMPs protected chondrocytes from the osteoarthritis environment. A) Schematic illustration of HNPs and HMPs protected chondrocytes from osteoarthritis environment. B) HIF‐1*α* gene, C) TIMP3 gene, D) MMP3 gene, E) MMP13 gene, F) Adamts‐5 gene, G) IL‐1*β* gene, H) IL‐6 gene, I) IL‐10 gene, J) TNF‐*α* gene, K) COX‐2 gene, L) quantification of ROS. The relative intensity of ROS was quantified by using an image pro plus 6.0 software. M) ROS changed in chondrocytes before and after being treated with HNPs/HMPs or NAC combined with HNPs/HMPs. It was found that the combination of NAC and HNPs/HMPs has no synergy on ROS scavenging as compared to pure NAC group and HNPs/HMPs treated groups. In the OA model, HNPs and HMPs distinctly promoted the expression of HIF‐1*α* and TIMP3, as well as significantly down regulated the osteoarthritis‐related genes as compared to IL‐1*β* treated group (MMP3, MMP13, Adamts‐5, IL‐1*β*, IL‐6, TNF‐*α*, and COX‐2). All the above experimental groups, except the CTR group, were incubated with IL‐1*β* to fabricate an OA chondrocyte model. The IL‐1*β* group was only incubated with IL‐1*β*, and the CTR group was untreated, the other groups were incubated with different concentrations of HNPs/HMPs for seven days after pretreating with IL‐1*β*. The relative gene amount of CTR groups was set as 1. NAC, an antioxidant, was used to inhibit inflammatory factors and ROS produce in chondrocytes. HNPs and HMPs significantly scavenged the production of ROS in chondrocytes. Repeat number: *n* = 6. In q‐PCR experiments, the statistical analysis between the IL‐1*β* group and the other groups was conducted. In ROS scavenged experiments, the statistical analysis between the Rosup group and the other groups was conducted. **p < 0.01, ***p < 0.001 (one‐way ANOVA followed by Dunnett's multiple comparisons test). Error bars represent mean ± SD.

To investigate the potential mechanism by which HNPs and HMPs protected chondrocytes from the inflammatory environment, the expression of inflammation related genes was measured (**Figure** **4**). First, IL‐1*β* was used to prepare an OA chondrocyte model. N‐acetyl‐L‐cysteine (NAC), a common antioxidant, was used to inhibit inflammatory factors and ROS production in chondrocytes. After incubating with different concentrations of HNPs or HMPs, OA chondrocytes were subjected to RT‐qPCR analysis. Compared with the IL‐1*β* group, HNP and HMP significantly inhibited the expression of ECM degeneration related genes (MMP3, MMP13, and Adamts‐5) and inflammatory factors (IL‐1*β*, IL‐6, TNF‐*α*, COX‐2), while the expression of IL‐10 (anti‐inflammatory factor) was elevated at the concentration of 3.125 mg ml^−1^ (Figure 4D–K). Interestingly, HNPs and HMPs had better effects on down‐regulation of inflammation related genes as compared with NAC (2 mM). Moreover, the gene expression of HIF‐1*α* and TIMP3 was distinctly increased after co‐culturing with HNPs and HMPs (Figure 4B,C). Additionally, the ROS scavenging effect of HNPs and HMPs was further investigated. Compared to the results observed in the positive control group (Rosup), HNPs and HMPs significantly inhibited ROS produced in chondrocytes within a concentration range of 0.049–3.125 mg ml^−1^ (Figure 4M). The quantification data showed that the residual ROS in the NAC group was similar to that in the group treated with HMPs at a concentration of 3.125 mg ml^−1^ (Figure 4L). Furthermore, the ROS scavenged capability of the combination of NAC and HNPs/HMPs was investigated. It was found that the combination of NAC and HNPs/HMPs did not show better ROS scavenging ability as compared to pure NAC groups or HNPs/HMPs treated groups, suggesting there was no synergistic effect.

### 2.5 HNPs and HMPs Stimulated Chondrocyte Maturation and rBMSC Osteogenic Differentiation

To investigate the stimulating effect on chondrocytes and rBMSCs, HNPs and HMPs were cocultured with chondrocytes and rBMSCs. CCK‐8 was employed to study the proliferation of chondrocytes and rBMSCs. The results indicated that HNPs and HMPs (0.049–3.125 mg ml^−1^) significantly promoted the proliferation of chondrocytes after 7 d of incubation (Figure S7A, Supporting Information). The proliferation of rBMSCs was enhanced by incubation with HNPs and HMPs at concentrations of 0.195–1.56 mg ml^−1^ and 0.049–0.195 mg ml^−1^, respectively (Figure S7B, Supporting Information). To further investigate cell proliferation, a cell cycle and apoptosis analysis kit was used to treat cellular samples, and then flow cytometry was employed to analyze the proliferation cells. In the cell cycle, DNA synthesis begins in the S phase, and the amount of DNA doubles in the G2M phase. Thus, cell proliferation can be proven by measuring the number of cells that stay in the S phase and the G2M phase. Compared to the CTR group, chondrocytes and rBMSCs treated with HNPs or HMPs possessed a high population of cells in the S phase or in the G2M phase, which indicated that HNPs and HMPs improved the proliferation capability of chondrocytes and rBSMCs within a concentration range of 0.049–1.56 mg ml^−1^ (Figure S7C,D, Supporting Information).

**Figure 5:**
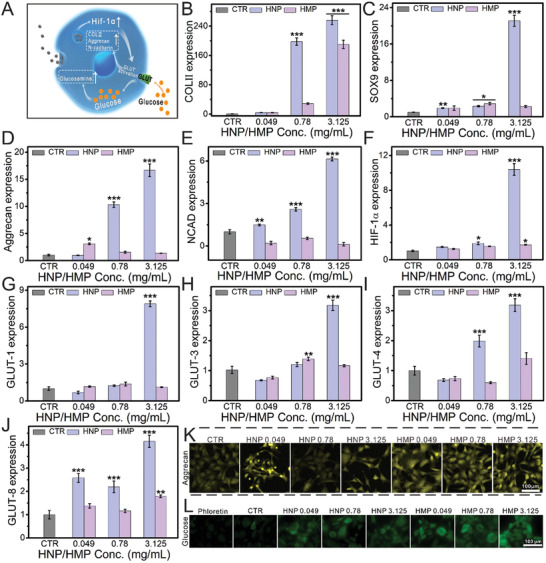
HNPs and HMPs stimulated the maturation of chondrocytes. A) Schematic illustration of HNPs stimulated chondrocyte maturation. After co‐culturing with different concentrations HNPs/HMPs for seven days, the relative genes B) COL IIs, C) SOX9, D) Aggrecan, E) NCAD, F) HIF‐1*α*, G) GLUT‐1, H) GLUT‐3, I) GLUT‐4, J) GLUT‐8 of chondrocyte maturation were elevated. K) The expression of Aggrecan protein in chondrocytes. L) Glucose uptake of chondrocytes cultured with HNPs/HMPs. HNPs and HMPs significantly promoted chondrocytes maturation via stimulating HIF‐1*α* and GLUT pathway related genes. The relative gene amount of CTR groups was set as 1. Repeat number: *n* = 6. In the experiment, the statistical analysis between the CTR group and the other groups was conducted, *p < 0.05, **p < 0.01, ***p < 0.001 (one‐way ANOVA followed by Dunnett's multiple comparisons test). Error bars represent mean ± SD.

Based on the good proliferative activity, the maturation of chondrocytes was investigated (**Figure** **5**). The gene expression of COL II, SOX9, aggrecan, and N‐cadherin (NCAD) was distinctly enhanced after culturing with HNPs (0.78–3.125 mg ml^−1^) for 7 d (Figure 5B–E). Additionally, the chondrocytes cultured with HMPs (3.125 mg mL^−1^) significantly expressed the COL II gene, and obviously enhanced the expression of SOX9 and aggrecan within a low concentration range. Immunofluorescence was used to further investigate the maturation of chondrocytes. CLSM images and quantification data showed that HNPs and HMPs significantly elevated the expression of aggrecan (Figure 5K and Figure S8A, Supporting Information) and COL II proteins in chondrocytes (Figure S9, Supporting Information).

To further elaborate the potential mechanism by which HNPs and HMPs promote chondrocyte maturation, the expression of GLUT‐1, GLUT‐3, GLUT‐4, and GLUT‐8 genes in the GLUT pathway was measured and the glucose uptake of chondrocytes was investigated (Figure 5G–J). Realtime quantitative polymerase chain reaction (RT‐qPCR) analysis demonstrated that the expression of GLUTs was obviously increased in the chondrocytes cultured with HNPs (0.049–3.125 mg ml^−1^). Additionally, HNPs and HMPs (3.125 mg ml^−1^) distinctly enhanced the expression of HIF‐1*α* in chondrocytes (Figure 5F). Importantly, CLSM images and flow cytometry showed that HNPs and HMPs distinctly promoted the glucose uptake in chondrocytes (Figure 5L and Figure S10, Supporting Information).

To further determine the stimulation effect of HNPs and HMPs on HIF‐1*α*, siRNA for HIF‐1*α* was used to pretreat chondrocytes. After pretreating with siRNA‐HIF‐1*α*, the expression of HIF‐1*α* in chondrocytes was obviously inactivated as compared to the CTR group. Whereas the expression of HIF‐1*α* was significantly increased in HNP and HMP groups as compared to the group only treated with siRNA‐HIF‐1*α* (Figure S11A, Supporting Information). Furthermore, the expression of COL II, SOX9, aggrecan, and NCAD, which are related to chondrocyte maturation, was significantly enhanced after co‐culturing with HNPs and HMPs. Additionally, the gene expression of GLUT‐1, GLUT‐3, GLUT‐4, and GLUT‐8 in the GLUT pathway was further enhanced in HNP and HMP groups (Figure S11, Supporting Information).

**Figure 6:**
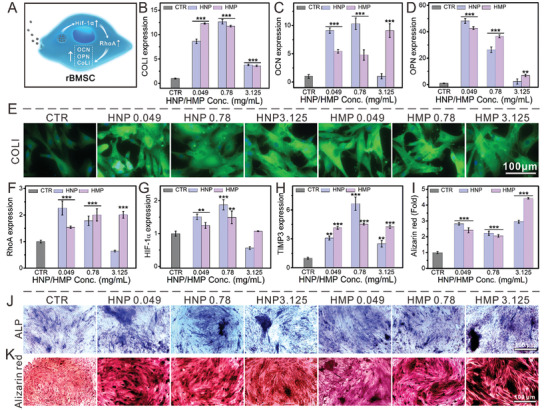
HNPs/HMPs accelerated the osteogenic differentiation of rBMSCs. A) Schematic illustration of HNPs and HMPs accelerated osteogenic differentiation. B) COL I gene, C) OCN gene, D) OPN gene. E) The expression of COL I protein in rBMSCs. F) RhoA gene, G) HIF‐1*α* gene, H) TIMP3 gene. Compared with the CTR group, the osteogenic differentiation of rBMSCs in HNP and HMP groups was significantly elevated at a certain concentration range after seven days. I) Alizarin red quantification for 21 days. J) and K) showed the ALP staining and Alizarin red staining of rBMSCs incubated with HNPs/HMPs for 14 days and 21 days, respectively. Compared with the CTR groups, relative ALP activity, and calcium deposits distinctly enhanced after rBMSCs incubating with HNPs/HMPs (0.049–3.125 mg ml^−1^) for 14 and 21 days, respectively. HNPs and HMPs accelerated osteogenic differentiation via stimulating RhoA mediated by HIF‐1*α*. The relative gene amount of CTR groups was set as 1. Repeat number: *n* = 6. In the experiment, the statistical analysis between the CTR group and the other groups was conducted, *p < 0.05, **p < 0.01, ***p < 0.001 (one‐way ANOVA followed by Dunnett's multiple comparisons test). Error bars represent mean ± SD.

Furthermore, osteogenic differentiation of rBMSCs was evaluated (**Figure** **6**A). RT‐qPCR results indicated that HNPs and HMPs significantly improved the expression of COL I, OCN, and OPN genes within the concentration range of 0.049–3.125 mg ml^−1^ (Figure 6B–D). CLSM images further showed that HNPs and HMPs distinctly stimulated the expression of COL I in rBMSCs (Figure 6E and Figure S8B, Supporting Information). To further evaluate the early marker and terminal marker of osteogenic differentiation, rBMSCs were cultured in HNPs and HMPs osteoinductive media for 14 d and 21 d, respectively. The results of alkaline phosphatase (ALP) activity and ALP staining showed that HNPs and HMPs significantly stimulated the expression of alkaline phosphatase in rBMSCs (Figure 6J, Figure S8C, Supporting Information). Alizarin red staining and quantification data showed that HNPs and HMPs distinctly promoted the formation of calcium nodules within a concentration of 0.049–3.125 mg ml^−1^ (Figure 6I,K). To further investigate the underlying mechanism by which HNPs and HMPs promote osteogenic differentiation, the expression of the RhoA, HIF‐1*α*, and TIMP3 genes was evaluated. RT‐qPCR results showed that HNPs and HMPs significantly stimulated the expression of RhoA and TIMP3 genes within a concentration range of 0.049–3.125 mg ml^−1^, whereas that of HIF‐1*α* was 0.049‐0.78 mg mL^−1^ (Figure 6F–H).

To further investigate the upregulated effect of HNPs and HMPs on RhoA, a GEF‐Rho GTPase inhibitor (Y16, 10 µM) was employed to inhibit the expression of RhoA in rBMSCs. As compared to the pure Y16 treated group, HNPs and HMPs distinctly enhanced the RhoA gene within a concentration range of 0.049–3.125 mg ml^−1^ (Figure S12A, Supporting Information). Furthermore, related genes of osteogenic differentiation, such as OCN and OPN, were significantly enhanced in rBMSCs (Figure S12E,F, Supporting Information). Additionally, TIMP3 and BMP2 were obviously elevated in the HMP group at a certain concentration of 3.125 mg ml^−1^, while HNPs significantly increased TIMP3 expressed at 0.049 mg ml^−1^(Figure S12C,D, Supporting Information).

### 2.6 HNP‐AKT Scaffolds Promoted the In Vivo Osteochondral Regeneration

**Figure 7:**
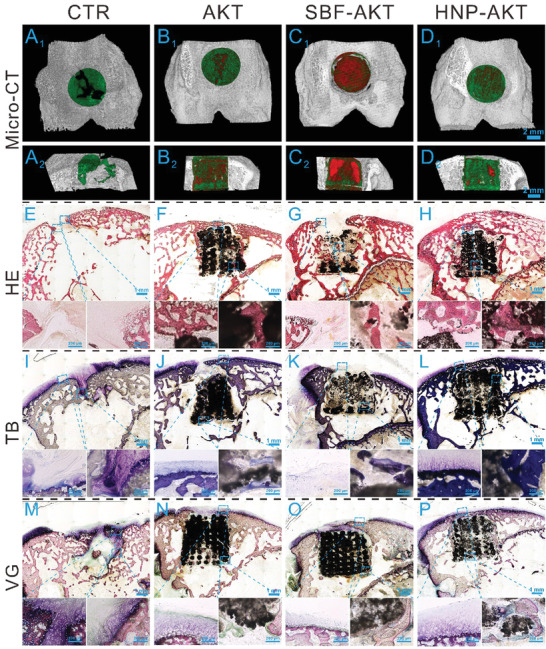
Osteochondral regeneration in vivo. A_1_–D_2_) showed the Micro‐CT images of the defects at 12 weeks post‐surgery. A_1_–D_1_) and A_2_–D_2_) displayed the transverse view and sagittal view of Micro‐CT images, respectively. The green color, red color, and grey‐white color in Micro‐CT images stand for new bone, scaffold, and native bone, respectively. Micro‐CT images indicated that HNP‐AKT group showed much more new bone tissue that than the other three experimental groups. E–H) HE staining, I–L) TB staining, M–P) VG staining. The scale bar in Micro‐CT images was 2 mm and the scale bars in the small images of HE, TB, and VG staining were 200 µm.

Based on the superior in vitro biological activities, HNP‐AKT scaffolds were selected to investigate the in vivo regeneration efficacy. An osteochondral defect model was fabricated in New Zealand white rabbits, and then scaffolds were implanted into the defect regions. Optical images of joint samples harvested at weeks 6 and 12 were taken (Figure S13, Supporting Information), and the corresponding micro‐CT images were collected (**Figure** **7** and Figure S14, Supporting Information). Overall, there was no inflammatory reaction in any of the samples. Glossy white tissue was found in the HNP‐AKT and CTR groups at 12 weeks, whereas large residual void spaces were found in the pure AKT and SBF‐AKT groups (Figure S13A_2_–D_2_, Supporting Information). The transverse view of micro‐CT images showed that much more neocalcified tissues existed in the HNP‐AKT group than in the AKT group, whereas a large vacancy remained in the CTR group. Barely calcified tissue was observed in the SBF‐AKT group at 12 weeks (Figure 7A_1_–D_1_). Sagittal views of 3D reconstruction micro‐CT images indicated that the neotissue was only generated in the margin and top surface of the defect region in the CTR group, whereas the neo‐bone tissue was abundant and well‐distributed in the HNP‐AKT group (Figure 7A_2_–D_2_). Furthermore, the bone mineral density (BMD) of the HNP‐AKT group was elevated as compared to that of the CTR and SBF‐AKT groups at 12 weeks (Figure S15B, Supporting Information). The relative bone volume fraction (BV/TV) and trabecular number (Tb. N) of the HNP‐AKT group displayed an increasing trend at 12 weeks compared to that of other groups (Figure S15C,D, Supporting Information).

To further investigate the hyaline cartilage and subchondral bone regenerative efficacy of HNP‐AKT scaffolds, histological analysis including H&E staining, TB staining, and improved Van Gieson's picrofuchsin (VG) staining was carried out. It further demonstrated that there was no inflammatory reaction after implantation of HNP‐AKT scaffolds for 6 weeks and 12 weeks (Figure 7 and Figure S14, Supporting Information). H&E staining images showed that there was a large vacant space with few tissues in the CTR and SBF‐AKT groups at 6 weeks, whereas the defect region in AKT and HNP‐AKT groups was covered with much more neotissue compared to that of the above two groups (Figure S14E_1_–H_3_, Supporting Information). The defect region in the HNP‐AKT group was well‐covered with a considerable amount of new bone tissue at 12 weeks, and there was a thin layer of neo‐bone on the top of the AKT scaffold, whereas the defect region in the CTR and SBF‐AKT groups showed no new bone tissue (Figure 7E–H). In TB staining, large amounts of glycosaminoglycans and well‐integrated hyaline cartilage were observed in the defect region of the HNP‐AKT group at week 12 (Figure 7L). However, hyaline cartilage in the defect region of the SBF‐AKT group was lacking, and few glycosaminoglycans were found in the CTR and AKT groups (Figure 7I–K). Furthermore, high magnification images showed that the orderly continuous osteochondral interface at the border zone was well‐integrated with the new hyaline cartilage and subchondral bone in the HNP‐AKT group at 12 weeks, which were similar to the original structure of natural osteochondral tissue (Figure S16, Supporting Information). In VG staining, the green color, hyacinthine color, and red color represent collagen fiber, hyaline cartilage, and bone tissue, respectively. These results indicated that organization was significantly improved and the presence of new cartilage and subchondral bone tissues in the HNP‐AKT group was significantly increased compared to other groups (Figure 7M–P, Figure S14M_1_–P_3_, Supporting Information). The new bone was well‐integrated into the HNP‐AKT scaffold, whereas the new tissue in the CTR, AKT, and SBF‐AKT groups was disordered and less abundant. To further investigate the tissue integration, images of the border zone between new and old tissue were acquired (Figure S17, Supporting Information). After 12 weeks of regeneration, new hyaline cartilage in the HNP‐AKT group nearly completely integrated with the surrounding cartilage, and the hyaline cartilage possessed abundant ECM and a large number of normal chondrocytes. The new tissue of the SBF‐AKT and AKT groups connected with old cartilage via the green collagen fiber. And the neotissue had fewer immature chondrocytes than the HNP‐AKT group had. Compared to the other three groups, the CTR group had a notable border between the old and new tissues. Based on the aforementioned staining results, the ICRS score was obtained (Figure S15A, Supporting Information). Compared to the score of the AKT and SBF groups, the HNP‐AKT group had the best score at both 6 weeks and 12 weeks. The results of histological analysis demonstrated that HNP‐AKT scaffolds distinctly facilitated the reconstruction of osteochondral tissue.

## 3 Discussion

Biochemical and physical microenvironments in OA need to be considered simultaneously for osteochondral regeneration. High levels of ROS and RNS could impede the natural regeneration process of osteochondral tissues in OA. It has been approved that ROS scavengers are effective for anti‐inflammatory therapeutics in OA. In the meanwhile, proper mechanical support is crucial for osteochondral regeneration due to the complex biomechanical microenvironment and anisotropic spatial architecture. Hence, tissue engineering scaffolds with antioxidative functions which could balance the biochemical and mechanical physical microenvironment might be the ideal material for osteochondral regeneration in OA. In this study, this synergistical effect could be achieved by designing the 3D printed bioceramic scaffold which was stably integrated with natural oxidants derived from hair. Our results indicated that the prepared scaffolds could scavenge a broad spectrum of free radicals that exist in OA, protect chondrocytes under the ROS microenvironment, and significantly enhance osteochondral regeneration in vivo. Consequently, the scaffolds integrated with HNPs/HMPs not only possessed superior ROS scavenging activity, but also provided proper mechanical support to balance the biochemical and physical microenvironment for cartilage and subchondral bone regeneration in OA.

Functionalization of bioceramic scaffolds with HNPs and HMPs requires mild reaction strategies in order to maintain the ROS scavenging activity and biological activities. For combining nano/micro particles with 3D scaffolds, hydrothermal methods or chemical methods involving organic solvents are commonly used. However, the main components of HNPs and HMPs are keratins and melanins, which could be easily denatured and lose bioactivity easily at high temperatures or in organic solvents. Thus, a gentle and green apatite mineralization method was employed to integrate HNPs and HMPs into 3D scaffolds. Compared to the high temperature used in the hydrothermal method, apatite mineralization was conveniently conducted in a body temperature environment (≈37 °C), which could maximally maintain the bioactivity of HNPs and HMPs. In addition, AKT scaffolds with well‐interconnected and highly uniform macropores were prepared through the 3D printing method. During the mineralization process, environmentally friendly SBF was used to accelerate mineralization and protect HNPs and HMPs from denaturation. As the HNPs and HMPs contained abundant functional groups and displayed electronegativity. The process of integrating HNPs and HMPs into 3D scaffolds may be interpreted as follows: First, during electrostatic gravitation, the electronegative HNPs/HMPs were bonded with the electropositive Ca^2+^ ions in scaffolds and attached to the scaffold surface.^[^ ^33^, ^34^ ^]^ Subsequently, abundant functional groups in HNPs/HMPs could adsorb Ca^2+^ and PO_4_^3−^ ions in SBF to form a Ca‐P and HNPs/HMPs composite surface in AKT scaffolds.^[^ ^35^, ^36^, ^37^ ^]^

In OA pathogenesis, multiple free radicals induce chondrocyte apoptosis, ECM degradation, cartilage degeneration, and subchondral bone dysfunction.^[^ ^38^, ^39^ ^]^ It is of great significance to scavenge a broad spectrum of radicals for efficient osteochondral regeneration in OA therapy. In this study, HNP‐AKT and HMP‐AKT scaffolds not only scavenged DPPH free radicals and hydroxyl radicals, but also exhibited SOD‐like and CAT‐like activities, which could obviously scavenge superoxide anions and hydrogen peroxide. With CAT‐like activity, the HNP‐AKT and HMP‐AKT scaffolds could significantly scavenge hydrogen peroxide, one of the byproducts of the SOD catalytic reaction, and generated oxygen for osteogenesis.^[^ ^40^ ^]^ Hence, HNP‐AKT and HMP‐AKT scaffolds with the appropriate mechanical properties are excellent candidates to regulate oxidative stress and recreate the healthy environment that is beneficial for osteochondral regeneration.

Interestingly, the scaffolds could distinctly stimulate the proliferation and maturation of chondrocytes due to the upregulation of HIF‐1*α* and GLUT pathway related genes by HNPs/HMPs. Previously, antioxidant has the capability to up‐regulate the expression of HIF‐1*α* and active the followed biological consequences.^[^ ^41^, ^42^ ^]^ The change of HIF‐1*α* influences the glyco‐metabolism intracellular, and glucose transporters help transport glucosamine and N‐acetyl glucosamine through the cell membrane to regulate inflammation.^[^ ^43^, ^44^ ^]^ Also, glucose transporters are involved in glycosaminoglycan synthesis.^[^ ^45^, ^46^ ^]^ In chondrocytes, endogenous glucosamine is derived from glucose metabolism and is a key raw material for synthesizing chondroitin sulfate. In the clinic, glucosamine is a common drug in OA therapy via improving glycosaminoglycan synthesis. However, the short retention and insufficient dose in joints hindered the therapeutic effect. In this study, HNPs and HMPs could stimulate the GLUT pathway to regulate glucosamine and enhance endogenous glycosaminoglycan synthesis to improve therapeutic effects for osteochondral regeneration. Our results showed that HNPs and HMPs significantly stimulated the expression of cartilage‐specific genes, including COL II, SOX9, aggrecan, and NCAD. After pretreating with siRNA‐HIF‐1*α*, the expression of HIF‐1*α* and GLUT pathway‐related genes, including GLUT‐1, GLUT‐3, GLUT‐4, and GLUT‐8, still could be significantly enhanced by HNPs and HMPs. The underlying mechanism of HNPs and HMPs stimulating the maturation of chondrocytes and reconstruction of hyaline cartilage may be elucidated as follows. In chondrocytes, HNPs and HMPs first enhanced the intracellular hypoxic environment, and then the expression of HIF‐1*α* was heightened. Subsequently, HIF‐1*α* mediated the expression of GLUT‐1 and GLUT‐3. The elevated GLUT significantly improved the expression of cartilage‐specific genes (COL II, SOX9, aggrecan, and NCAD) and promoted the synthesis of glycosaminoglycan and ECM (such as aggrecan and COL II proteins). Furthermore, GLUT could also promote the transportation of glucose, thus providing energy for cell activity and providing important materials for chondroitin sulfate production in chondrocytes. Hence, HNP‐AKT and HMP‐AKT scaffolds could stimulate chondrocyte maturation and cartilage repair due to the upregulation of HIF‐1*α* and enhancement of the GLUT pathway via HNPs and HMPs.

## 4 Conclusion

In this study, a novel antioxidative bioceramic scaffold (HNP/HMP‐AKT) with ROS scavenging and osteochondral regeneration capabilities was successfully demonstrated to simultaneously balance the biochemical and physical microenvironment in OA. Importantly, the bioscaffold could distinctly stimulate the proliferation and maturation of chondrocytes due to the stimulation of the GLUT pathway via HNPs/HMPs. The aforementioned results suggested that this bioscaffold with a broad ROS scavenging spectrum and osteochondral regeneration activity could meet the demands for simultaneous anti‐inflammation and rehabilitation, offering a smart strategy for OA treatment.

## 5 Experimental Section

### Extraction and Characterization of HNPs and HMPs

HMPs were isolated from human hair by using an alkaline extraction method. Human hair used in this study was contributed by the volunteers from the group. This experiment was approved by the Medical Ethics Committee, Shanghai East Hospital, School of Medicine, Tongji University. In brief, 2 g of hair was added to 1 M at 60 °C in NaOH (Sinopharm Group Co. Ltd., China) under slight stirring. Subsequently, the extract was dialyzed and centrifuged at 2000 rpm to obtain HMPs. HNPs were obtained by HMP ultrasonication. Finally, HNPs and HMPs were purified in ultrapure water (UPW) and lyophilized for further reaction. The transmission electron microscopy (TEM) images of HNPs and HMPs were obtained from a JEM‐2100F transmission electron microscope. FT‐IR detection was conducted under a Perkin‐Elmer Spectrum Two FT‐IR spectrophotometer. The particle sizes of HNPs and HMPs were measured by a Malvern (Nano‐ZS90) dynamic light scattering particle sizer. Ultraviolet and visible absorption spectra were recorded on a Cary 60 UV‐Vis spectrophotometer (Agilent Technologies, USA).

### Antioxidant Effect of HNPs and HMPs

To investigate the nitrogen free radical scavenging capability of HNPs and HMPs, diphenylpicrylhydrazyl (DPPH) free radicals were used. Briefly, 2 mg of DPPH was dissolved in 48 ml of dehydrated alcohol to prepare the DPPH working solution. Then, HNPs/HMPs were added to the DPPH working solution and incubated at 37 °C in a shaking bath for 30 min. Subsequently, photographs were taken and the UV‐vis spectrum was recorded. Furthermore, the superoxide dismutase (SOD)‐like activity of HNPs and HMPs was investigated by using a total SOD enzyme assay kit (A001‐3, Nanjing Jiancheng Bioengineering Institute, China).

Next, the catalase (CAT)‐like activity of HNPs and HMPs was evaluated via two methods. Method one: a CAT assay kit (A007‐1) produced by Nanjing Jiancheng Bioengineering Institute was used to measure the CAT‐like activity of HNPs and HMPs. Method two: according to the principle of the CAT catalytic hydrogen peroxide (H_2_O_2_, Sinopharm Group Co. Ltd, China) production of oxygen, a portable dissolved oxygen tester (JPB‐607A, Leici, Shanghai Yidian Scientific Instrument Co., Ltd, China) was employed to measure the CAT‐like activity of HNPs and HMPs indirectly. The concentration of H_2_O_2_ chosen to investigate the CAT‐like activity of HNPs and HMPs was 10 mM. In brief, HNPs/HMPs and H_2_O_2_ were added to 10 ml of anaerobic UPW under slow stirring. The dissolved oxygen was recorded by a portable dissolved oxygen tester.

Based on the aforementioned studies, the hydroxyl radical scavenging activity of HNPs and HMPs was evaluated. First of all, the hydroxyl radical was generated through the Fenton reaction of 5 mM H_2_O_2_ and 1.8 mM FeSO_4_ (Sinopharm Group Co. Ltd., China) at 37 °C for 10 min. Subsequently, HNPs/HMPs were added into the hydroxyl radical solution. After allowing a reaction for 30 min, the amount of hydroxyl radicals remaining was evaluated by measuring the characteristic absorbance at 510 nm. The characteristic absorbance was generated by reacting 1.8 mM salicylic acid (SA, Macklin, China) with residual hydroxyl radicals. Photographs of the reacted solutions were captured by a digital camera.

### Preparation of HNP‐AKT Scaffolds and HMP‐AKT Scaffolds

A 3D printed biomaterial formulation containing 5 g of AKT powder, 0.14 g of sodium alginate, and 2.6 g of 20.0 wt% F‐127 was prepared to fabricate AKT scaffolds. The biomaterial formulation was extruded through a 0.22 mm nozzle by using a GeSiM 3D printer to obtain the primary scaffolds. Then, the primary scaffolds were dried overnight and calcined at 1350°C to obtain pure AKT scaffolds. To maintain the bioactivity of HNPs and HMPs, the apatite mineralization method was employed to prepare HNP‐AKT scaffolds and HMP‐AKT scaffolds. Briefly, 50 mg of HNPs/HMPs were dispersed in 10 ml of simulated body fluid (SBF, pH 7.4) to obtain the HNP/HMP working solution, respectively. The exact concentrations of the electrolytes and ions present in the Simulated Body Fluid are as follow: Na^+^ 142.0 mM, K^+^ 5.0 mM, Mg^2+^ 1.5 mM, Ca^2+^ 2.5 mM, Cl^−^ 103.0 mM, HCO_3_^−^ 10.0 mM, HPO_4_^2−^ 1.0 mM, SO_4_^2−^ 0.5 mM. Subsequently, the HNP/HMP working solution was transferred into a 15 ml centrifuge tube containing approximately 1.0 g of pure AKT scaffolds and placed in a 37 °C constant temperature shaking bath with a speed of 120 rpm to obtain HNP‐AKT or HMP‐AKT scaffolds, respectively. Pure AKT scaffolds treated with pure SBF under the same conditions became SBF‐AKT scaffolds. Pure AKT scaffolds and SBF‐AKT scaffolds were used as controls. The Simulated Body Fluid is a metastable solution, with the composition of sodium chloride, potassium chloride, dipotassium hydrogen phosphate, magnesium chloride, calcium chloride, tris, and sodium bicarbonate, especially supersaturated calcium ions and phosphate ions.

### Characterization of HNP‐AKT scaffolds and HMP‐AKT scaffolds

Photographs and scanning electron microscopy (SEM) images of AKT scaffolds before and after modification with HNPs/HMPs were obtained from a digital camera and Hitachi S‐4800 SEM (Tokyo, Japan), respectively. The FT‐IR spectrum of the scaffolds was recorded by a Perkin‐Elmer Spectrum Two FT‐IR spectrophotometer. To conduct a mechanical test, cylindrical scaffolds with a height of 10.0 ± 0.705 mm and a diameter of 8.0 ± 0.685 mm were prepared. Scaffolds were subjected to mechanical testing (1.00 mm min^−1^) via using an INSTRON 5566 (Germany) universal mechanical test machine. To investigate degradation properties, scaffolds were treated with Tris–HCl solution for 1, 3, 7, 14, 21, and 35 d. The Tris–HCl solution volume to scaffold mass was 200 ml g^−1^. The Tris–HCl solution was refreshed and harvested at the set time point. Then, an inductively coupled plasma atomic emission spectroscopy (ICPAES, 710ES, Varian, USA) was employed to measure the Ca, Mg, and Si levels in the Tris–HCl. After treatment with Tris–HCl, scaffolds were dried for 12 h. Then, the degradation rate of scaffolds was accurately measured. Additionally, the release of HNPs and HMPs from scaffolds was studied in UPW. HNP‐AKT and HMP‐AKT scaffolds were soaked in UPW for 2–20 d in a 37 °C constant temperature shaking bath. The UPW volume used with the scaffolds was 1 m l per scaffold. The UPW was refreshed and harvested at different time points. Subsequently, a detergent compatible Bradford protein assay kit (P0006C, Beyotime, China) was used to measure concentrations of HNPs and HMPs in the UPW. All experiments were performed in sextuplicate.

### Antioxidant Capability of HNP‐AKT Scaffolds and HMP‐AKT Scaffolds

To evaluate the antioxidant capability of the antioxidative scaffolds, the nitrogen free radical and hydroxyl radical scavenging activities, as well as the SOD‐like and CAT‐like activities were studied. To investigate the nitrogen free radical scavenging capability of scaffolds, the DPPH working solution was prepared as the aforementioned method, and then scaffolds were immersed into DPPH working solution for 2–120 h. Digital photographs and UV‐vis spectra of the DPPH working solution before and after reacting with the scaffolds were recorded at the set time points. The SOD‐like activity of the scaffolds was evaluated by employing a total SOD enzyme assay kit. Briefly, the SOD working solution was prepared following the manufacturer's protocol, and then scaffolds reacted with the SOD working solution at 37 °C for 1 h. After the SOD working solution was separated from the scaffolds, 2 ml of chromogenic reagent was added into the working solution at room temperature for 10 min. Finally, the absorbance at 550 nm of the final solution was measured by a UV‐vis spectrophotometer. The CAT‐like activity of HNP‐AKT scaffolds and HMP‐AKT scaffolds was evaluated by using a CAT assay kit (A007‐1, Nanjing Jiancheng Bioengineering Institute, China) and a portable dissolved oxygen tester. CAT working solution was prepared following the manufacturer's protocol, and then scaffolds reacted with the CAT working solution at 37 °C. At the set time points, the working solution was separated from the scaffolds, and then ammonium molybdenum acid was used as a chromogenic reagent to evaluate the residual H_2_O_2_. Subsequently, the absorbance at 405 nm of the final solution was measured via a UV‐vis spectrophotometer. Furthermore, 25 mM of H_2_O_2_ was used to verify the CAT‐like activity of HNP‐AKT scaffolds and the HMP‐AKT scaffolds. Briefly, a scaffold and H_2_O_2_ were added into 10 ml of anaerobic UPW water under slow stirring, and the dissolved oxygen was recorded by a portable dissolved oxygen tester. Finally, oxygen bubbles generated on the surface of the scaffolds were captured after reaction for 20 min.

### Cell Culture of Chondrocytes and rBMSCs

Primary chondrocytes were isolated from 3 weeks old New Zealand White rabbits. Chondrocytes were cultured in Dulbecco's modified Eagle's medium (C11885500BT, Gibco, Thermo Fisher Scientific) which contained 10% fetal calf serum (10099141, Gibco, Thermo Fisher Scientific) and 1% penicillin‐streptomycin (15140122, Gibco, Thermo Fisher Scientific). rBMSCs (RBXMX‐01001) were purchased from Cyagen Biosciences and cultured in a special mesenchymal stem cell basal medium (RBXMX‐9001, Cyagen Biosciences, USA).

### Cell Proliferation Assay

Cell proliferation of chondrocytes and rBMSCs cultured with HNPs/HMPs was studied by a CCK‐8 (CK04, Dojindo, Japan) assay. Briefly, the aforementioned cells were seeded into 96‐well plates (2 × 10^3^ cells per well) and incubated with different concentrations of HNPs/HMPs for 1–7 d. Then, the cells were incubated with CCK‐8 working solution for 2 h, and the absorbance was measured at 450 nm. In order to display the proliferation of cells visually, the absorbance values at different time points were used directly. Furthermore, flow cytometry was further used to investigate the proliferation of rBMSCs and chondrocytes. After chondrocytes and rBMSCs were incubated with HNPs/HMPs for 3 d, cell cycle and apoptosis analysis kit (C1052, Beyotime, China) were employed to investigate cell cycles, and a Flow Jo 3.0 software was used to analyze the data. The different concentrations of HNPs/HMPs were prepared as follows: 50 mg of dried HNPs or HMPs was dispersed in the cell culture medium to make 16 ml (3.125 mg ml^−1^). Then, the obtained HNP/HMP solutions were diluted to 1/2 (1.56 mg ml^−1^), 1/4 (0.78 mg ml^−1^), 1/8 (0.39 mg ml^−1^), 1/16 (0.195 mg ml^−1^), 1/32 (0.098 mg ml^−1^), and 1/64 (0.049 mg ml^−1^).

### Protection of OA Chondrocytes In Vitro

To investigate the related mechanism of HNP and HMP protected chondrocytes, the HIF pathway and inflammation‐related genes were evaluated. First, IL‐1*β* (10 ng ml^−1^) was used to prepare an OA chondrocyte model in vitro. After incubating with different concentrations of HNPs/HMPs for 7 d, OA chondrocytes were subjected to RT‐qPCR analysis following the aforementioned method. The OA related genes (MMP3, MMP13, Adamts‐5, IL‐1*β*, IL‐6, IL‐10, TNF‐*α*, and COX‐2) and HIF pathway related genes (HIF‐1*α* and TIMP3) were measured. To investigate the ROS scavenging capability of HNPs and HMPs in chondrocytes, a DCFH‐DA probe (S0033, Beyotime, China) was used. Before treatment with Rosup solution, 2 mM of NAC, or different concentrations of HNPs/HMPs combined with/without NAC (2 mM) were incubated with chondrocytes for 24 h. Subsequently, the chondrocytes were incubated with a DCFH‐DA probe according to the manufacturer's protocol. Finally, fluograms were captured by an EVOS FL Auto imaging system, and quantitative data were obtained from an Image‐Pro Plus software. NAC, which could inhibit inflammatory factors and ROS production in chondrocytes, was used as the positive control group. The Rosup group was only treated with a compound mixture from the ROS assay kit, while the CTR group was untreated.

### Differentiation of Chondrocytes and rBMSCs

Based on the superior proliferative activity of cells, chondrocyte maturation and rBMSC osteogenic differentiation were investigated via quantitative real‐time transcriptase polymerase chain reaction (RT‐ qPCR). After 7 d of incubation with HNPs/HMPs, the total RNA of cell samples was extracted and reverse transcribed by using a ToYoBo RNAprep Micro Kit (FSK 201, ToYoBo, Japan). Subsequently, the cDNA was subjected to RT‐qPCR via using a SYBR Green qPCR Master Mix (QPK‐201, ToYoBo, Japan) and a Light Cycler apparatus (Step One Plus Real‐Time PCR system, Thermofisher, USA). After operating the RT‐qPCR cycle conditions, a 2^−ΔΔ^ *^Ct^* method was used to calculate target gene expression. The target genes in chondrocytes were COL II, SOX9, aggrecan, NCAD, HIF‐1*α*, glucose transporter (GLUT)‐1, GLUT‐3, GLUT‐4, and GLUT‐8. The target genes in rBMSCs were COL I, OCN, OPN, RhoA, HIF‐1*α*, and TIPM3. In RT‐qPCR analysis, GAPDH was employed as a reference gene, and the gene expression of the CTR group was set as 1. All experiments in this part were performed in sextuplicate. All the primer sequences used in PCR experiments were prepared via Invitrogen OLIGO 7.0 software. The primer sequences are shown in Table S1, Supporting Information.

Furthermore, specific proteins involved in chondrogenesis and osteogenesis were investigated via an immunofluorescence technique. In brief, 4% paraformaldehyde was used to fix cellular samples after incubation with HNPs/HMPs for 3 d. Subsequently, the cellular samples were treated with a primary antibody (aggrecan: Abcam ab3773; COL II: Abcam ab3092; COL I: Abcam ab6308) and a second antibody (Abcam ab150105 or Abcam 175472). Then, DAPI was used to observe nuclei. Finally, argon laser lines of 405 nm, 488 nm, and 568 nm were used to capture the confocal laser scanning microscopy (CLSM) images. The quantitative data were obtained from Image‐Pro Plus software.

Additionally, alkaline phosphatase (ALP) activity and calcium nodules in the osteogenesis process were further investigated. After incubating in osteogenic induction medium with different concentrations of HNPs/HMPs for 14 d, an ALP assay kit (P0321, Beyotime, China) and BCIP/NBT phosphatase color development kit (C3206, Beyotime, China) were used to quantify and identify the expression of ALP in rBMSCs. The absorbance data and digital photographs were obtained from a multifunction microplate reader (ELx808, BioTek, USA) and an optical microscope (Optika, Italy), respectively. To investigate the deposition of calcium nodules generated in rBMSCs, alizarin red staining was processed after incubation with osteogenic induction medium with different concentrations of HNPs/HMPs for 21 d. In brief, the alizarin red staining solution (Cyagen Biosciences, USA) was utilized to incubate the glutaraldehyde‐fixed rBMSCs, and the calcium nodules were observed via an optical microscope. Subsequently, the cellular samples were treated with 100 mM cetylpyridinium chloride (Macklin, China) and the absorbance was measured at 570 nm. In this part, experiments were conducted in sextuplicate. The HNP/HMP osteogenic induction medium was prepared as follows: 50 mg of dried HNPs or HMPs was dispersed in osteogenic induction medium to make 16 ml (3.125 mg ml^−1^). Then, the obtained solution was diluted to 1/4 (0.78 mg ml^−1^), and 1/64 (0.049 mg ml^−1^). The rBMSCs cultured with osteogenic induction medium without HNPs and HMPs were used as a control group.

### The Promotion of HIF‐1*α* and RhoA

To further determine the stimulation of HIF‐1*α* in chondrocytes and induction of RhoA in rBMSCs, siRNA for HIF‐1*α* and Y16 for RhoA were used to pretreat with cellular samples, respectively. In brief, 100 pmol siRNA (Gene Pharma, China) and 5 µl lipo6000 (C0526, Beyotime, China) were used to pretreat chondrocytes for 24 h before incubating with HNPs/HMPs for 7 d. The sequences of the siRNA for HIF‐1*α* were as followed. Sense sequence: GGGCCGUUCAAUUUAUGAATT. Antisense sequence: UUCAUAAAUUGAACGGCCCTT. Also, 10 µM of Y16 was used to pretreat with rBMSCs before co‐culturing with HNPs/HMPs for 7 d. Subsequently, total RNA of cell samples was extracted and RT‐ qPCR was conducted. The target genes in chondrocytes were HIF‐1*α*, COL II, SOX9, aggrecan, NCAD, GLUT‐1, GLUT‐3, GLUT‐4, and GLUT‐8. The target genes in rBMSCs were RhoA, HIF‐1*α*, TIPM3, BMP2, OCN, and OPN. GAPDH was employed as a reference gene, and the gene expression of the blank control group (CTR) or Y16 group was set as 1. All experiments in this part were performed in sextuplicate.

### Glucose Uptake of Chondrocytes In Vitro

To study whether HNPs and HMPs could facilitate the glucose uptake in chondrocytes, a glucose uptake assay kit (ab204702, Abcam, Britain) was used. After incubating chondrocytes with HNPs/HMPs for 3 d, a glucose uptake mix was used to culture the chondrocytes for 30 min. Subsequently, glucose uptake in chondrocytes was observed under an EVOS FL Auto imaging system (Life Technologies, Thermo Fisher Scientific, USA). The quantitative data were obtained from Image‐Pro Plus software. Moreover, flow cytometry detection was further used to investigate glucose uptake. After treatment with HNPs/HMPs and glucose uptake mix, chondrocytes were collected, and flow cytometric analysis was conducted. Phloretin is a natural polyphenol that can inhibit glucose transport by suppressing GLUT‐1, SGLT1, and SGLT2 activity. In this study, phloretin was used as a control to inhibit glucose transport by suppressing GLUT‐1 activity.

### Adhesion and Proliferation of Chondrocytes and rBMSCs on Scaffolds

Ultraviolet radiation was applied to sterilize scaffolds before the experiment. Scaffolds were then inoculated with chondrocytes and rBMSCs (0.5 × 10^4^ per well) after being transferred into 48‐well plates, respectively. At different time points (1 d, 3 d, and 7 d), samples were transferred into new 48‐well plates and incubated with CCK‐8 working solution for 2 h. Subsequently, the absorbance of the solution was evaluated at 450 nm. In addition, chondrocytes and rBMSCs (5.0 × 10^4^ per well) were incubated with scaffolds for 4 h, 12 h, and 24 h to evaluate cell adhesion and cell morphology. At the set time points, scaffolds were transferred to a new 48‐well plate and incubated with the CCK‐8 working solution for 2 h. Subsequently, the absorbance was detected and the adhesion rate was calculated. Before SEM and CLSM analyses were performed, cells were subjected to the following protocols. In brief, 4% paraformaldehyde was used to anchor cellular samples, and then graded ethanol was used to dehydrate cellular samples. For CLSM analysis, diamidinophenylindole (DAPI, Sigma‐Aldrich, USA) and rhodamine‐phalloidin (Amyjet Scientific, Wuhan, China) were used to stain nuclei and cytoskeletons. The distribution and microfilaments of cells were observed via a Leica confocal laser scanning microscope. All experiments in this part were performed in sextuplicate.

### In Vivo Osteochondral Regeneration

All rabbits were treated according to the guidelines of the Institutional Animal Care and Use Committee of Tongji University. Twenty‐four adult rabbits (≈2.5 kg) were used to fabricate an osteochondral defect model (diameter: 6 mm, height: 6 mm). Then, scaffolds were implanted into the defect region with a top surface level that was the same as the tidemark. The defects treated with nothing served as the blank control group (CTR, *n* = 6), and the other defects were transplanted with HNP‐AKT scaffolds (*n* = 6), or implanted with SBF‐AKT scaffolds (*n* = 6), or embedded with AKT scaffolds (n = 6). At week 6 and week 12, knee samples were collected for osteochondral regeneration evaluation. After knee samples were treated with paraformaldehyde for 48 h, a micro‐CT (Skyscan 1172, Bruker, Germany) was used to obtain relative bone information. A Data Viewer and CTAn software were applied in the data analysis process. Additionally, a CT vox short cut software was used to obtain the transverse view and sagittal view of images. Hematoxylin eosin (H&E, Beijing Solarbio Science & Technology Co., Ltd.), improved Van Gieson's picrofuchsin (VG), and toluidine blue (TB) staining were used to evaluate the osteochondral regeneration effect. Six hard tissue slices in each group were used in each histological staining experiment. Finally, an EVOS FL Auto imaging system was employed to capture the optical images. Six investigators were blinded to the International Cartilage Repair Society (ICRS) score.

### Statistical Analysis

In this study, all data statistical analyses were conducted to use the software of SPSS 22.0 and showed as mean ± SD. Results are representative of two independent experiments. Statistical parameters for each experiment were displayed in relevant figure legends. The value of *n* stands for the number of replicates, and *P* stands for the probability value. Furthermore, the information of the sample size and the probability value was displayed in figure legends. The *P*‐value less than 0.05 was considered statistically significant. The data were demonstrated with * for 0.01 < *p* < 0.05, ** for 0.001 < *p* < 0.01, and *** for *p* < 0.001.

## Conflict of Interest

The authors declare no conflict of interest.

## Author Contributions

C. D. performed the experiments, data analyses and wrote the manuscript. Y. C., C. D., and C. W. contributed to the conception of this study and improved the manuscript. Q. Z. and X. W. contributed to natural antioxidative nanomaterials extraction and scaffolds fabrication. M. Z., C. X., and Q. F. helped conducted the in vivo experiments. T. L. helped conducted Micro‐CT analysis. H. C. contributed to antioxidant capability study of the scaffolds. F. Y. contributed to the experimental design and performed the analysis with constructive discussions.

## Supporting information

Supporting Information

## Data Availability

The data that support the findings of this study are available from the corresponding author upon reasonable request.

## References

[1] W. S. Choi , G. Lee , W. H. Song , J. T. Koh , J. Yang , J. S. Kwak , H. E. Kim , S. K. Kim , Y. O. Son , H. Nam , I. Jin , Z. Y. Park , J. Kim , I. Y. Park , J. I. Hong , H. A. Kim , C. H. Chun , J. H. Ryu , J. S. Chun , Nature 2019, 566, 254. 30728500 10.1038/s41586-019-0920-1

[2] J. Sellam , F. Berenbaum , Nat. Rev. Rheumatol. 2010, 6, 625. 20924410 10.1038/nrrheum.2010.159

[3] B. Sharma , S. Fermanian , M. Gibson , S. Unterman , D. A. Herzka , B. Cascio , J. Coburn , A. Y. Hui , N. Marcus , G. E. Gold , Sci. Transl. Med. 2013, 5, 167ra6. 10.1126/scitranslmed.3004838 PMC3972413 23303605

[4] R. K. Das , V. Gocheva , R. Hammink , O. F. Zouani , A. E. Rowan , Nat. Mater. 2016, 15, 318. 26618883 10.1038/nmat4483

[5] H. Kwon , W. E. Brown , C. A. Lee , D. Wang , N. Paschos , J. C. Hu , K. A. Athanasiou , Nat. Rev. Rheumatol. 2019, 15, 550. 31296933 10.1038/s41584-019-0255-1 PMC7192556

[6] C. Mandrycky , Z. Wang , K. Kim , D.‐H. Kim , Biotechnol. Adv. 2016, 34, 422. 26724184 10.1016/j.biotechadv.2015.12.011 PMC4879088

[7] C. M. Madl , S. C. Heilshorn , H. M. Blau , Nature 2018, 557, 335. 29769665 10.1038/s41586-018-0089-z PMC6773426

[8] F. Gao , Z. Xu , Q. Liang , H. Li , L. Peng , M. Wu , X. Zhao , X. Cui , C. Ruan , W. Liu , Adv. Sci. 2019, 6, 1900867. 10.1002/advs.201900867 PMC6685475 31406678

[9] X. Hu , Y. Wang , Y. Tan , J. Wang , H. Liu , Y. Wang , S. Yang , M. Shi , S. Zhao , Y. Zhang , Adv. Mater. 2017, 29, 1605235. 10.1002/adma.201605235 28185322

[10] H. Zhang , H. Huang , G. Hao , Y. Zhang , H. Ding , Z. Fan , L. Sun , Adv. Funct. Mater. 2021, 31, 2006697.

[11] C. Deng , R. Lin , M. Zhang , C. Qin , Q. Yao , L. Wang , J. Chang , C. Wu , Adv. Funct. Mater. 2019, 29, 1806068.

[12] L. Chen , C. Deng , J. Li , Q. Yao , J. Chang , L. Wang , C. Wu , Biomaterials 2019, 196, 138. 29643002 10.1016/j.biomaterials.2018.04.005

[13] J. A. Bolduc , J. A. Collins , R. F. Loeser , FreeRadic. Biol. Med. 2019, 132, 73. 10.1016/j.freeradbiomed.2018.08.038 PMC6342625 30176344

[14] D. Richard , Z. Liu , J. X. Cao , A. M. Kiapour , J. Willen , S. Yarlagadda , E. Jagoda , V. B. Kolachalama , J. T. Sieker , G. H. Chang , P. Muthuirulan , M. Young , A. Masson , J. Konrad , S. Hosseinzadeh , D. E. Maridas , V. Rosen , R. Krawetz , N. Roach , T. D. Capellini , Cell 2020, 181, 362. 32220312 10.1016/j.cell.2020.02.057 PMC7179902

[15] S. Li , F. Ma , X. Pang , B. Tang , L. Lin , Carbohyd. Polym. 2019, 212, 387. 10.1016/j.carbpol.2019.02.061 30832871

[16] L. I. Rachek , V. I. Grishko , S. P. LeDoux , G. L. Wilson , Free Radic. Biol. Med. 2006, 40, 754. 16520228 10.1016/j.freeradbiomed.2005.09.028

[17] R. B. Zhou , A. S. Yazdi , P. Menu , J. Tschopp , Nature 2011, 469, 221. 21124315 10.1038/nature09663

[18] D. Li , G. R. Xie , W. C. Wang , Am. J. Med. Sci. 2012, 344, 486. 22885622 10.1097/MAJ.0b013e3182579dc6

[19] H. D. Lim , Y. S. Kim , S. H. Ko , I. J. Yoon , S. G. Cho , Y. H. Chun , B. J. Choi , E. C. Kim , J. Pineal Res. 2012, 53, 225. 22507555 10.1111/j.1600-079X.2012.00991.x

[20] R. Schreck , P. Rieber , P. A. Baeuerle , EMBO J. 1991, 10, 2247. 2065663 10.1002/j.1460-2075.1991.tb07761.x PMC452914

[21] T. Yin , L. Yang , Y. Liu , X. Zhou , J. Sun , J. Liu , Acta Biomater. 2015, 25, 172. 26143603 10.1016/j.actbio.2015.06.035

[22] J. Li , J. Zhang , Y. Chen , N. Kawazoe , G. Chen , ACS Appl. Mater. Interfaces 2017, 9, 35683. 28944661 10.1021/acsami.7b12486

[23] G. Zhong , X. Yang , X. Jiang , A. Kumar , H. Long , J. Xie , L. Zheng , J. Zhao , Nanoscale 2019, 11, 11605. 31173033 10.1039/c9nr03060c PMC6776464

[24] D. W. Zheng , S. Hong , L. Xu , C. X. Li , K. Li , S. X. Cheng , X. Z. Zhang , Adv. Mater. 2018, 30, 1800836. 10.1002/adma.201800836 29782675

[25] M. B. Rahmany , R. R. Hantgan , M. Van Dyke , Biomaterials 2013, 34, 2492. 23332318 10.1016/j.biomaterials.2012.12.008

[26] X. Bao , J. Zhao , J. Sun , M. Hu , X. Yang , ACS Nano 2018, 12, 8882. 30028940 10.1021/acsnano.8b04022

[27] T. Sun , D. Jiang , Z. T. Rosenkrans , E. B. Ehlerding , D. Ni , C. Qi , C. J. Kutyreff , T. E. Barnhart , J. W. Engle , P. Huang , Adv. Funct. Mater. 2019, 29, 1904833. 10.1002/adfm.201904833 PMC7017599 32055240

[28] S. Hong , Q.‐L. Zhang , D.‐W. Zheng , C. Zhang , Y. Zhang , J.‐J. Ye , H. Cheng , X.‐Z. Zhang , iScience 2020, 23, 100778. 31901818 10.1016/j.isci.2019.100778 PMC6948237

[29] C. Feng , W. Zhang , C. Deng , G. Li , J. Chang , Z. Zhang , X. Jiang , C. Wu , Adv. Sci. 2017, 4, 1700401. 10.1002/advs.201700401 PMC5737106 29270348

[30] T. Li , D. Zhai , B. Ma , J. Xue , P. Zhao , J. Chang , M. Gelinsky , C. Wu , Adv. Sci. 2019, 6, 1901146. 10.1002/advs.201901146 PMC6774059 31592134

[31] V. Afonso , R. Champy , D. Mitrovic , P. Collin , A. Lomri , Jt., Bone, Spine 2007, 74, 324. 10.1016/j.jbspin.2007.02.002 17590367

[32] M. Xu , D. Zhai , L. Xia , H. Li , S. Chen , B. Fang , J. Chang , C. Wu , Nanoscale 2016, 8, 13790. 27380634 10.1039/c6nr01952h

[33] W. C. Fang , H. Zhang , J. W. Yin , B. G. Yang , Y. B. Zhang , J. J. Li , F. L. Yao , Cryst. Growth Des. 2016, 16, 1247.

[34] L. B. Mao , H. L. Gao , H. B. Yao , L. Liu , H. Colfen , G. Liu , S. M. Chen , S. K. Li , Y. X. Yan , Y. Y. Liu , S. H. Yu , Science 2016, 354, 107. 27540008 10.1126/science.aaf8991

[35] C. T. Wu , J. A. Chang , J. Y. Wang , S. Y. Ni , W. Y. Zhai , Biomaterials 2005, 26, 2925. 15603787 10.1016/j.biomaterials.2004.09.019

[36] J. D. Hartgerink , E. Beniash , S. I. Stupp , Science 2001, 294, 1684. 11721046 10.1126/science.1063187

[37] Y. Liu , S. A. Liu , D. Luo , Z. J. Xue , X. A. Yang , L. Cu , Y. H. Zhou , T. Wang , Adv. Mater. 2016, 28, 8740. 27530607 10.1002/adma.201602628

[38] Y. Henrotin , B. Kurz , T. Aigner , Osteoarthritis Cartilage 2005, 13, 643. 15936958 10.1016/j.joca.2005.04.002

[39] J. H. Zhong , T. Scholz , A. C. Y. Yau , S. Guerard , U. Huffmeier , H. Burkhardt , R. Holmdahl , Sci. Adv. 2018, 4, eaas9864. 29774240 10.1126/sciadv.aas9864 PMC5955621

[40] S. Stegen , I. Stockmans , K. Moermans , B. Thienpont , P. H. Maxwell , P. Carmeliet , G. Carmeliet , Nat. Commun. 2018, 9, 2557. 29967369 10.1038/s41467-018-04679-7 PMC6028485

[41] P. Gao , H. Zhang , R. Dinavahi , F. Li , Y. Xiang , V. Raman , Z. M. Bhujwalla , D. W. Felsher , L. Cheng , J. Pevsner , L. A. Lee , G. L. Semenza , C. V. Dang , Cancer Cell 2007, 12, 230. 17785204 10.1016/j.ccr.2007.08.004 PMC2084208

[42] P. T. Schumacker , Cancer Cell 2015, 27, 156. 25670075 10.1016/j.ccell.2015.01.007

[43] J. L. Frey , D. P. Stonko , M.‐C. Faugere , R. C. Riddle , Bone Res. 2014, 2, 14005. 26273518 10.1038/boneres.2014.5 PMC4472139

[44] T. Cramer , Y. Yamanishi , B. E. Clausen , I. Forster , R. Pawlinski , N. Mackman , V. H. Haase , R. Jaenisch , M. Corr , V. Nizet , G. S. Firestein , H. P. Gerber , N. Ferrara , R. S. Johnson , Cell 2003, 112, 645. 12628185 10.1016/s0092-8674(03)00154-5 PMC4480774

[45] S. Y. Lee , E. D. Abel , F. X. Long , Nat. Commun. 2018, 9, 4831. 30446646 10.1038/s41467-018-07316-5 PMC6240091

[46] C. A. Thaiss , M. Levy , I. Grosheva , D. P. Zheng , E. Soffer , E. Blacher , S. Braverman , A. C. Tengeler , O. Barak , M. Elazar , R. Ben‐Zeev , D. Lehavi‐Regev , M. N. Katz , M. Pevsner‐Fischer , A. Gertler , Z. Halpern , A. Harmelin , S. Aamar , P. Serradas , A. Grosfeld , H. Shapiro , B. Geiger , E. Elinav , Science 2018, 359, 1376. 29519916 10.1126/science.aar3318

