# Supplementary material for: Bioceramic Scaffolds with Antioxidative Functions for ROS Scavenging and Osteochondral Regeneration
Source: Adv Sci (Weinh). 2022 Feb 19;9(12):2105727. doi: 10.1002/advs.202105727 (PMC9036007; doi:10.1002/advs.202105727)
Supplement: Supplementary file 1 — Supporting Information [file ADVS-9-2105727-s001.pdf]

ADVANCED SCIENCE
Open Access

Supporting Information

for *Adv. Sci.*, DOI: 10.1002/advs.202105727

Bioceramic Scaffolds with Antioxidative Functions for ROS Scavenging and Osteochondral Regeneration

Cuijun Deng, Quan Zhou, Meng Zhang, Tian Li, Haotian Chen, Chang Xu, Qishuai Feng, Xin Wang, FengYin, Yu Cheng*,Chengtie Wu**

Supporting Information for

Bioceramic Scaffolds with Antioxidative Functions for ROS Scavenging and Osteochondral Regeneration

Cuijun Deng[a,b,c], Quan Zhou[a,b], Meng Zhang[b], Tian Li[b], Haotian Chen[a,c], Chang Xu[a], Qishuai Feng[a], Xin Wang[b], Feng Yin[c,]*, Yu Cheng[a,]*, Chengtie Wu[b,]*.

[a] Translational Medical Center for Stem Cell Therapy & Institute for Regenerative Medicine, Shanghai East Hospital, Tongji University School of Medicine, 1800 Yuntai Road, Shanghai 200123, P.R. China

[b] State Key Laboratory of High Performance Ceramics and Superfine Microstructure, Shanghai Institute of Ceramics, Chinese Academy of Sciences. Shanghai 200050, P.R. China

[c] Department of Joint Surgery, Shanghai East Hospital, School of Medicine, Tongji University, Shanghai 200123, P.R. China.

*** Corresponding Author**

Email: 001yinfeng@sina.com (F. Yin), yucheng@tongji.edu.cn (Y. Cheng), chengtiewu@mail.sic.ac.cn (C. Wu)

Table S1. The primer sequences used for RT-qPCR analysis

Gene	Forward primer	Reverse primer
GAPDH	5-TCACCATCTTCCAGGAGCGA	5-CACAATGCCGAAGTGGTCGT
COL II	5-AACACTGCCAACGTCCAGAT	5-CTGCAGCACGGTATAGGTGA
SOX9	5-GGTGCTCAAGGGCTACGACT	5-GGGTGGTCTTTCTTGTGCTG
Aggrecan	5-AGGTCGTGGTGAAAGGTGTTG	5-GTAGGTTCTCACGCCAGGGA
NCAD	5-TCATCTTCGTTTCCATTGGA	5-TAAGAACTCTGTAAGTTTTGGC
HIF-1α	5- CCATGTGACCATGAGGAAAT	5- CGGCTAGTTAGGGTACACTT
GLUT-1	5- GCCCTGCATGTCCTATCTGA	5- TGAAATTCGAGGTCCAGTTGG
GLUT-3	5- CGTCATCTTTGCCGTCTTC	5- ACATGGGTGGTGGTCTCAA
GLUT-4	5- GGCGGCATGATTTCCTCC	5- GAAGGGCAGCAGGATCAGCT
GLUT-8	5- CACGTCAAGGGTGTGGCT	5- CAGGGACGCAGAACAAAGTG
MMP3	5-ATGGACCTTCTTCAGC AA	5-TCATTATGTCAGCCTCTC
MMP13	5-AGGAGCATGGCGAC TTCTAC	5-TAAAAACAGCTCCGCATCAA
Adamts-5	5-TGTCC TGCCAGCGGATGT	5-ACGGAATTACTGTACGGCCTACA
IL-1β	5- CAGGACCTGGACCTCTGCTGTC	5- GAGCCACAACGACTGACAAGACC
IL-6	5- GAAAACACCAGGGTCAGCAT	5-CAGCCACTGGTTTTTCTGCT
IL-10	5-GAGAACCACAGTCCAGCCAT	5-CATGGCTTTGTAGACGCCTT
TNF-α	5- CTCCTACCCGAACAAGGTCA	5- CGGTCACCCTTCTCCAACT
COX-2	5-TTGACCAGTACAAGTGCGAC	5-AGTGCGTAAGGATGTAGTGC
TIMP3	5- CATCCACACGGAAGCCTCTGA	5- TTACAGCCCAGGTGGTAGCG
COL I	5-CTTCTGGCCCTGCTGGAAAGGATG	5-CCCGGATACAGGTTTCGCCAGTAG
BMP2	5-CGCCTCAAATCCAGCTGTAAG	5-GGGCCACAATCCAGTCGTT
OCN	5-CCGGGAGCAGTGTGAGCTTA	5-AGGCGGTCTTCAAGCCA TACT
OPN	5-CACCATGAGAATCGCCGT	5-CGTGACTTTGGGTTTCTACGC
RhoA	5-CCCAACGTGCCTATCATCTT	5-GCAGCTCTAGTGGCCATTTC

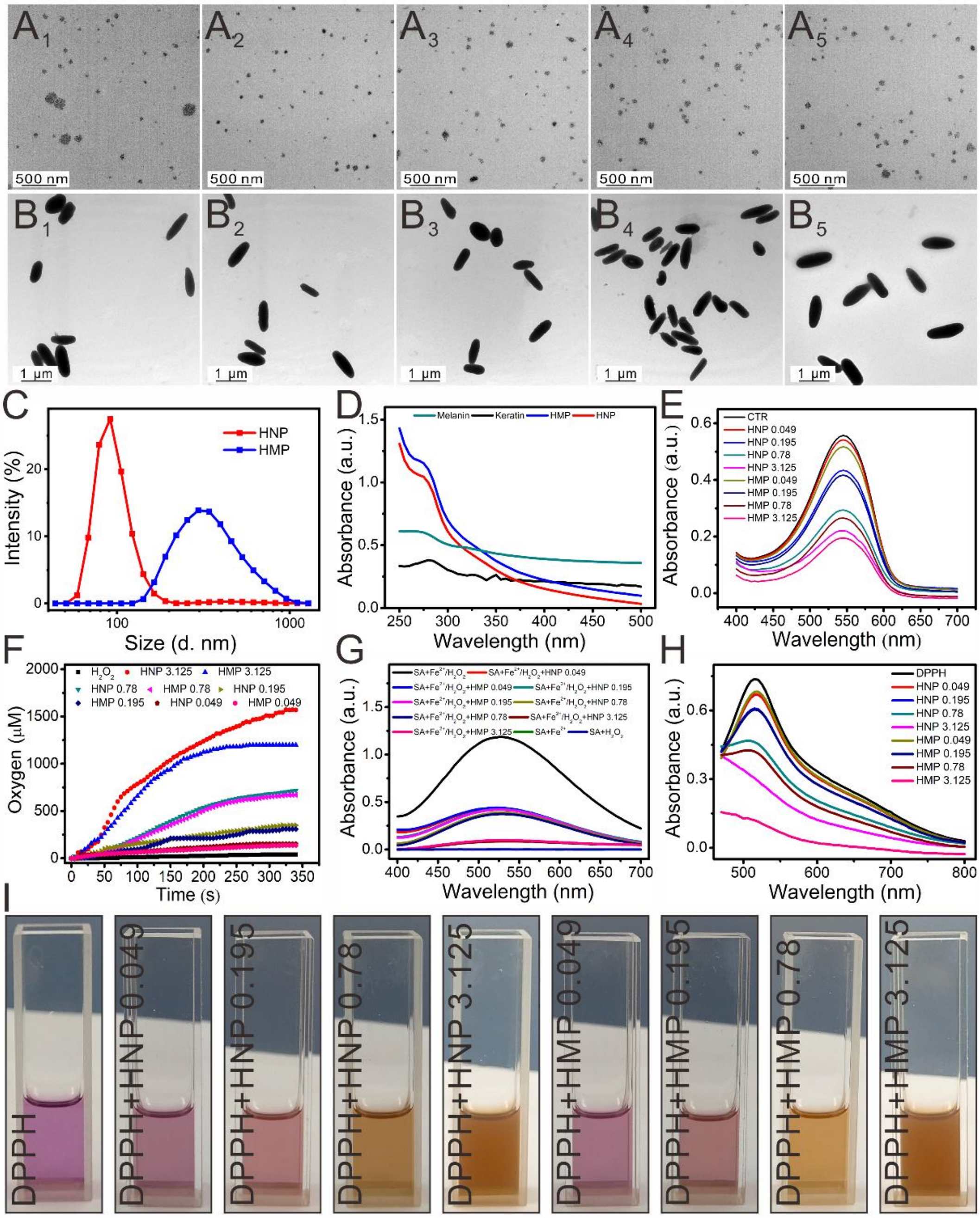

Figure S1. Characterization and free radicals scavenging capability of HNPs and HMPs. (A_{1-5}) TEM images of HNPs. (B_{1-5}) TEM images of HMPs. The size of both HNPs and HMPs was similar in different pools. (C) Particle size distribution of HNPs and HMPs. (D) UV-vis spectrum of HNPs and HMPs. Compared to the ultraviolet region, the absorbance value of HNPs and HMPs in the visible region was lower. (E) The superoxide radical scavenging effect of HNPs and HMPs. (F) O_2 production from the H_2O_2 solution (10 mM) with vs without HNPs/HMPs. CAT-like and SOD-like activities were increased with the concentration increase of HNPs and HMPs. (G) The UV-vis absorbance spectrum of SA post reaction with Fe^{2+}/H_2O_2 with and without the presence of HNPs/HMPs. (H) The UV-vis spectrum of DPPH free radical after reacting with HNPs or HMPs. (I) The color of DPPH free radical after reacting with different concentrations of HNPs and HMPs.

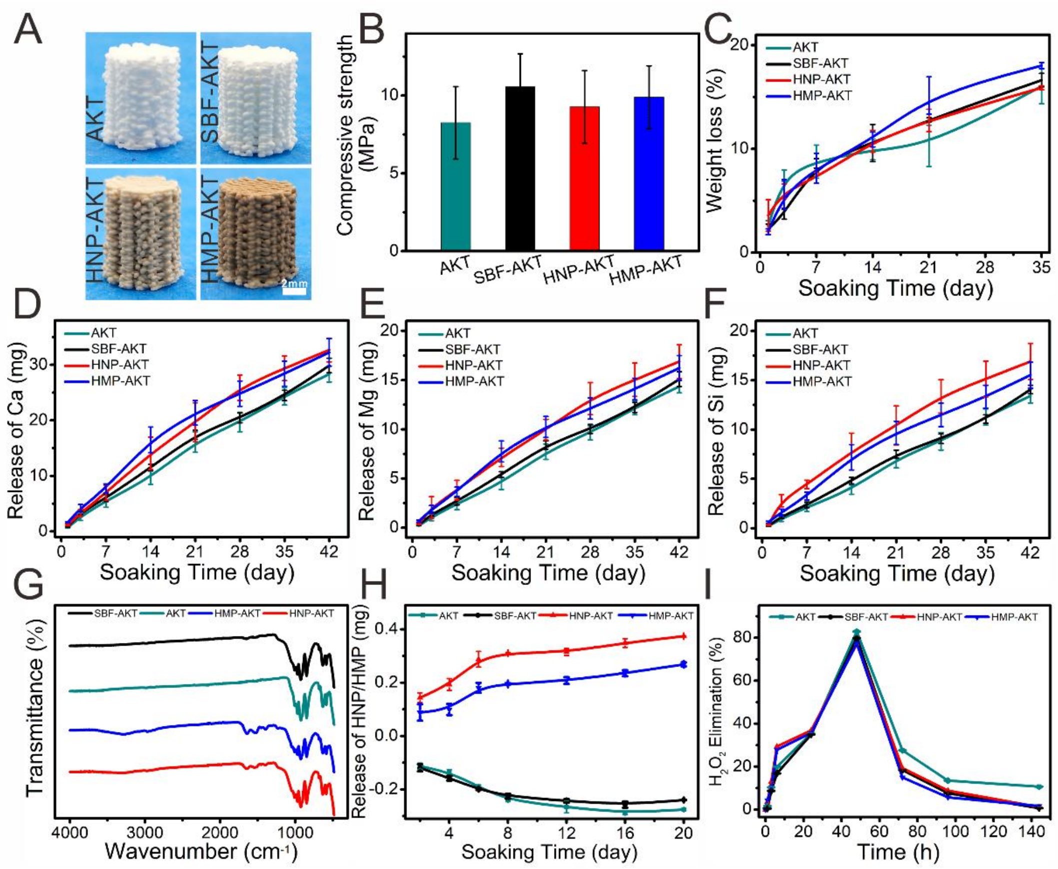

Figure S2. Characterization of AKT scaffolds before and after modifying with HNPs and HMPs. Figure (A) is the digital photographs of scaffolds used for compressive strength test. (B) Compressive strength. There has no significant difference between the four experimental groups. (C) Weight loss of the scaffolds before and after treating with SBF, HNP or HMP. The released of Ca (D), Mg (E) and Si (F) has no significant difference between the four experimental groups. (G) FT-IR spectrum of the scaffolds. The FT-IR spectrum and color changed of the scaffolds demonstrated that HNP and HMP successfully coated on the surface of AKT scaffold. (H) Release of HNPs and HMPs. (I) H_2O_2 scavenging effects. As compared to AKT scaffolds, the H_2O_2 scavenging efficiency of HNP-AKT and HMP-AKT scaffolds has an upward trend within the first 6 h, while this advantage cannot be maintained for a long time. Repeat number: n = 6. In H_2O_2 scavenging experiments, two-way ANOVA followed by Dunnett's multiple comparisons test was used, and the results showed no significant difference between AKT group and the other groups.

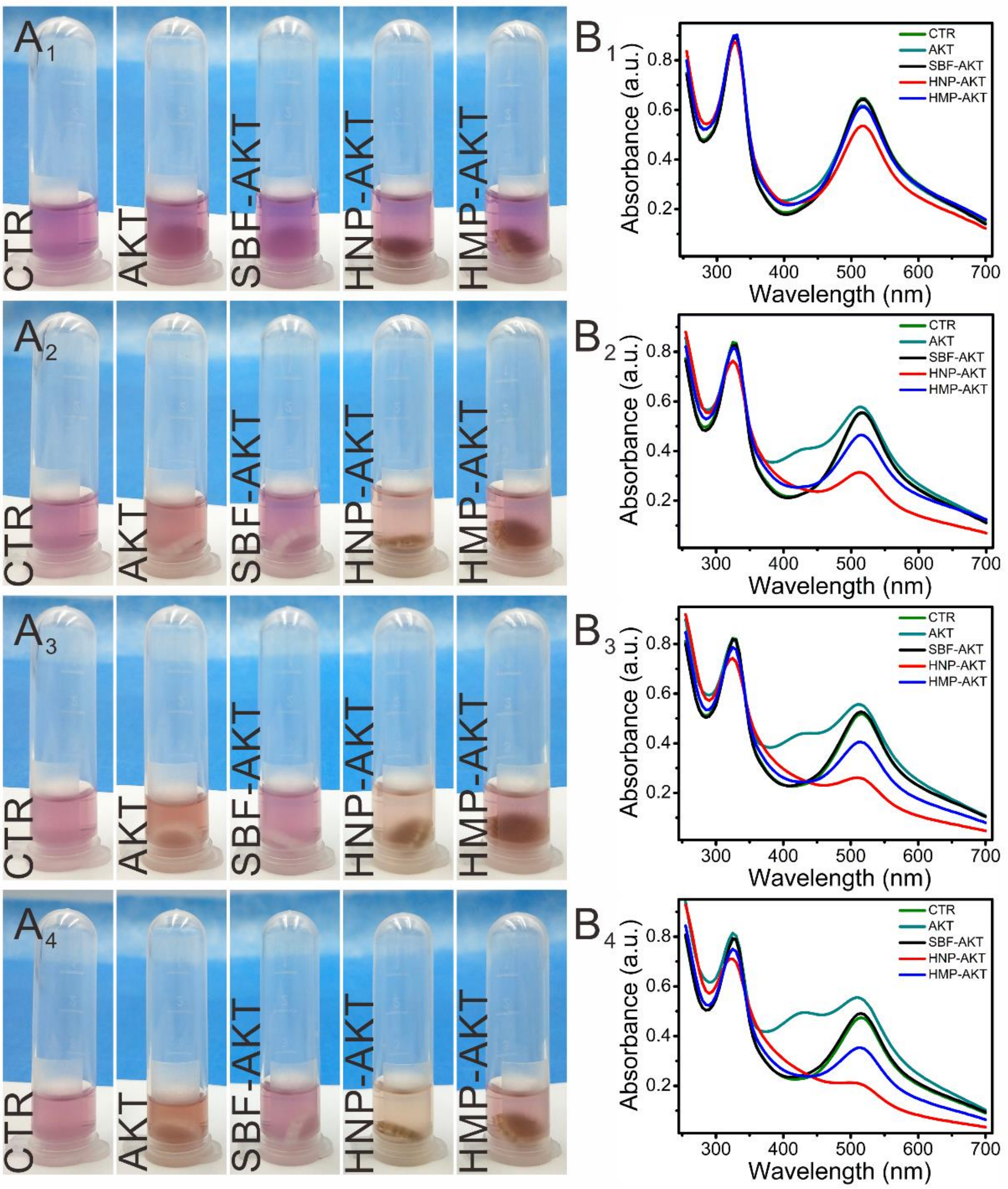

Figure S3. Nitrogen free radical scavenging capability of AKT scaffolds before and after modifying. (A_1-A_4) Digital photographs of DPPH free radical solution before and after treating with the scaffolds. (A_1) 2 h, (A_2) 12 h, (A_3) 18 h, (A_4) 24 h. (B_1-B_4) The UV-vis absorbance spectrum of DPPH free radical solution post reaction with the scaffolds for different periods of time. (B_1) 2 h, (B_2) 12 h, (B_3) 18 h, (B_4) 24 h. After coating with HNPs/HMPs, AKT scaffolds significantly scavenged DPPH free radical as compared with SBF-AKT scaffold and pure AKT scaffold.

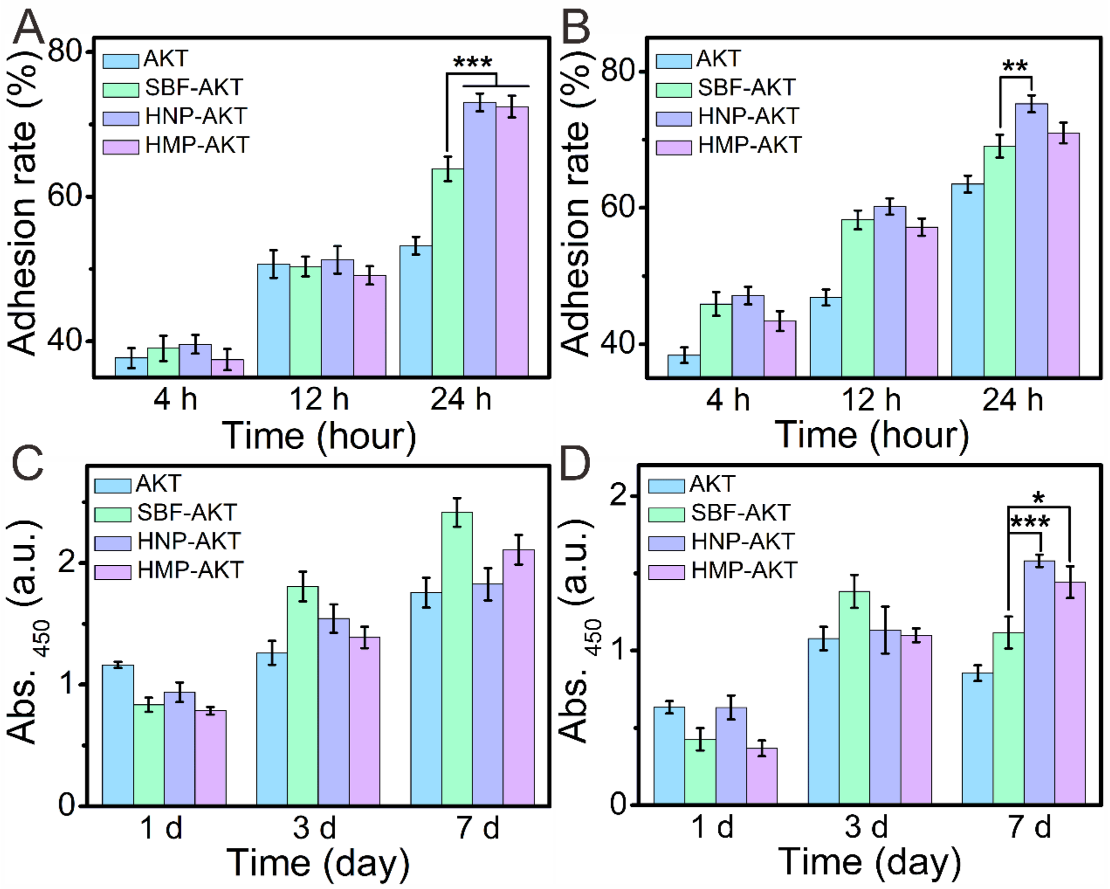

Figure S4. Adhesion and proliferation of chondrocytes and rBMSCs in the scaffolds. (A) and (C) showed the adhesion and proliferation of chondrocytes cultured in the scaffolds, respectively. (B) and (D) displayed the adhesion and proliferation of rBMSCs cultured in the scaffolds, respectively. After 24 h of incubation, HNP-AKT scaffold possessed much more chondrocytes and rBMSCs attached on the surface of scaffold as compared to that of SBF-AKT scaffold. Furthermore, HNP-AKT and HMP-AKT scaffolds sustainably stimulating the proliferation of chondrocytes and rBMSCs in 7 days. Repeat number: n=6. The statistical analysis between SBF-AKT group and the other groups was conducted at the time points of 24 h and 7 d in adhesion experiment and proliferation experiment, respectively (one-way ANOVA followed by Dunnett's multiple comparisons test). *$p < 0.05$, **$p < 0.01$, ***$p < 0.001$, Error bars represent mean±SD.

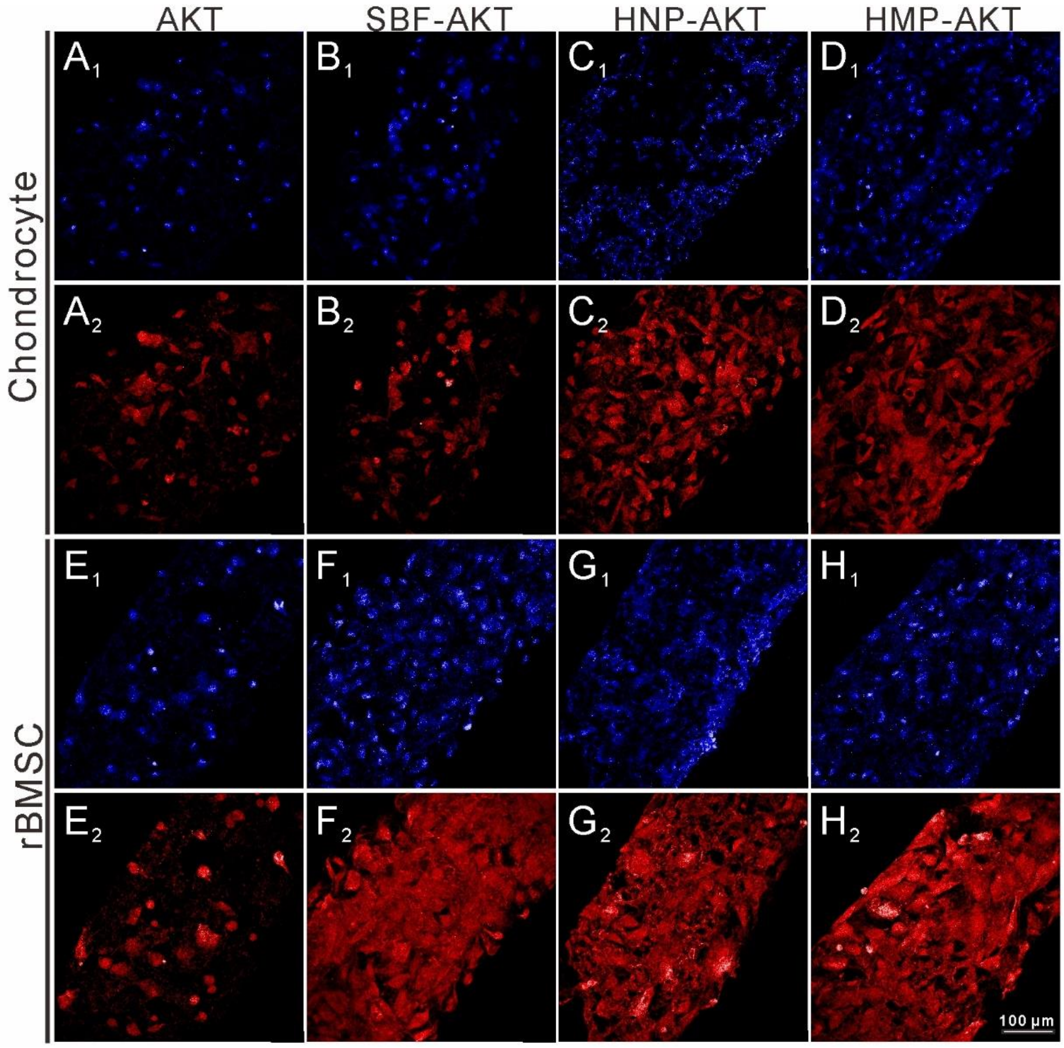

Figure S5. CLSM images of chondrocytes and rBMSCs on the scaffolds. (A_1-D_1) and (E_1-H_1) showed the DAPI staining of the cell nucleus in chondrocytes and rBMSCs, respectively. (A_2-D_2) and (E_2-H_2) displayed the cytoskeletons of chondrocytes and rBMSCs, respectively.

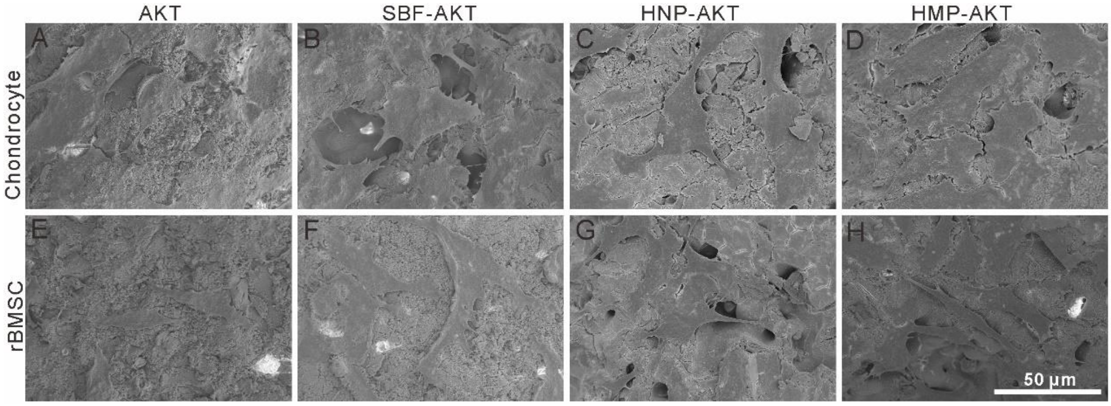

Figure S6. Cell morphology of chondrocytes and rBMSCs on the surface of the scaffold. (A) and (E) AKT scaffolds, (B) and (F) SBF-AKT scaffolds, (C) and (G) HNP-AKT scaffolds, (D) and (H) HMP-AKT scaffolds.

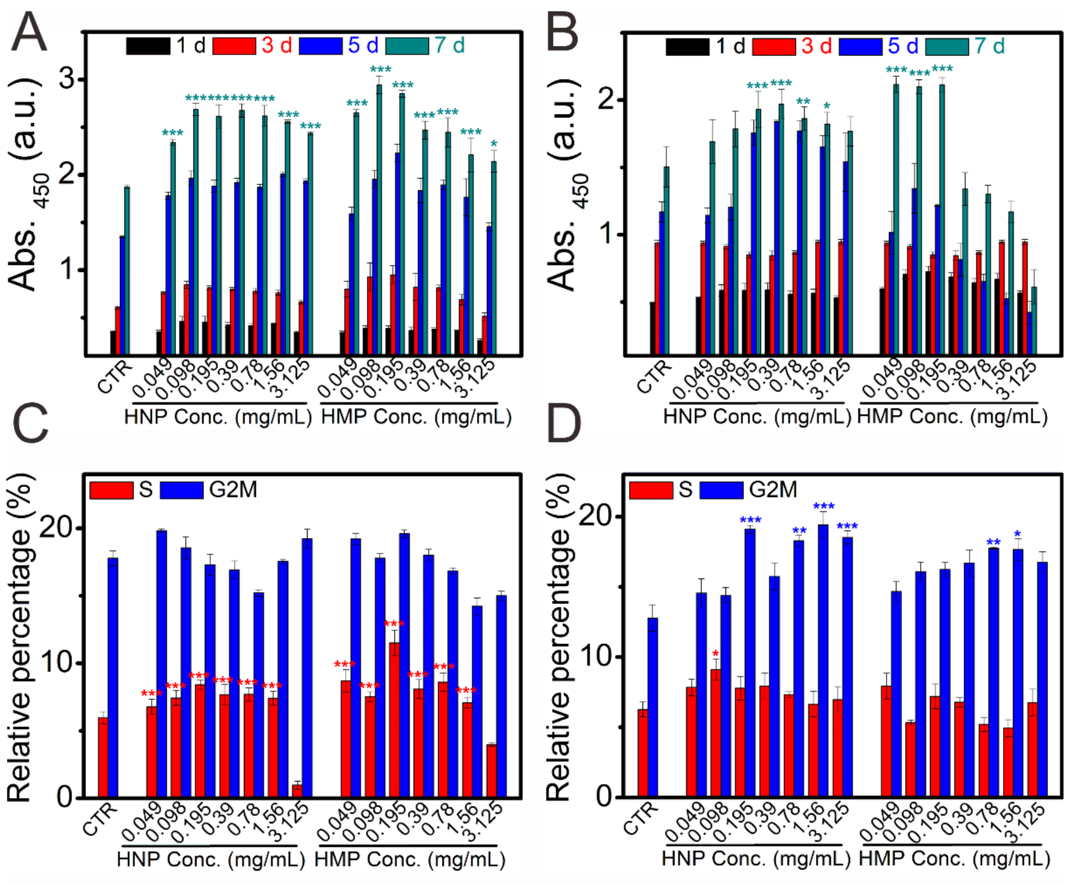

Figure S7. The proliferation of chondrocytes and rBMSCs cultured with HNPs and HMPs. (A) Proliferation of chondrocytes. (B) Proliferation of rBMSCs. Within a concentration range of 0.049-3.125 mg mL^{-1}, HNPs and HMPs significantly promoted the proliferation of chondrocytes after 7 days of incubating. HNPs distinctly enhanced the proliferation of rBMSCs within a concentration range of 0.195-1.562 mg mL^{-1}, while HMPs stimulated rBMSCs proliferation within a concentration range of 0.049-0.195 mg mL^{-1}. (C) The relative percentage of S and G2M phases' chondrocytes after incubating with HNPs and HMPs for 3 days. (D) The relative percentage of S and G2M phases' rBMSCs after incubating with HNPs and HMPs for 3 days. As compared with the CTR group, chondrocytes and rBMSCs treated with HNPs or HMPs possessed much more cells in S phase or G2M phase, which indicated HNPs and HMPs elevated the proliferation capability of chondrocytes and rBSMCs within the concentration range of 0.049-1.562 mg mL^{-1}. Repeat number: n=6. In the experiment, the statistical analysis between CTR group and the other groups was conducted, *$p < 0.05$, **$p < 0.01$, ***$p < 0.001$ (one-way ANOVA followed by Dunnett's multiple comparisons test). Error bars represent mean±SD.

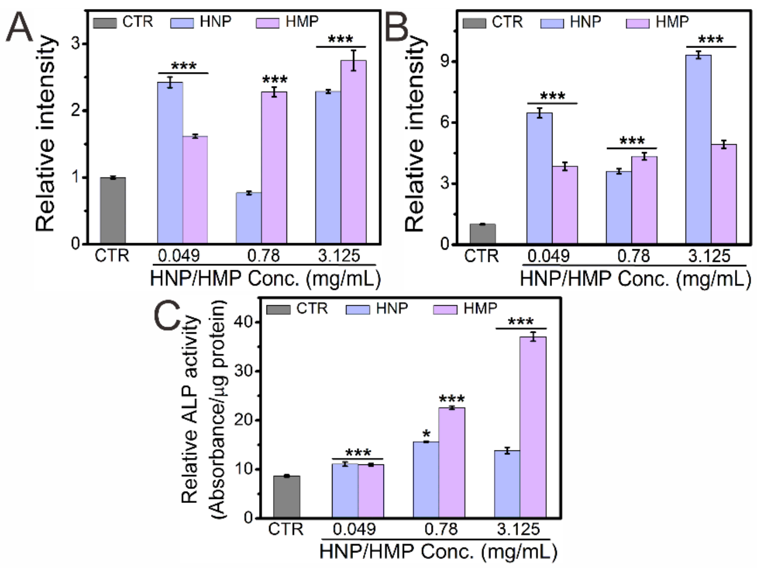

Figure S8. Quantification of Aggrecan, COL I, and ALP activity. (A) Aggrecan in chondrocytes. (B) COL I in rBMSCs. (C) ALP activity of rBMSCs incubated with HNPs/HMPs for 14 days. The relative intensity of Aggrecan and COL I was quantified by using an image pro plus 6.0 software. Repeat number: n=6. In the experiment, the statistical analysis between CTR group and the other groups was conducted, *p < 0.05, ***p < 0.001 (one-way ANOVA followed by Dunnett's multiple comparisons test). Error bars represent mean±SD.

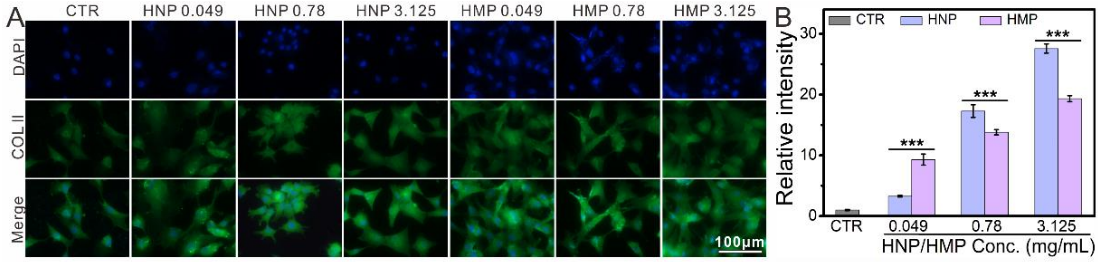

Figure S9. COL II expressed in chondrocytes co-culturing with HNPs and HMPs for 3 d. (A) CLSM images of COL II protein, (B) COL II quantification by using image-pro plus software. Repeat number: n = 6. In the experiment, the statistical analysis between CTR group and the other groups was conducted, ***p < 0.001 (one-way ANOVA followed by Dunnett's multiple comparisons test). Error bars represent mean±SD.

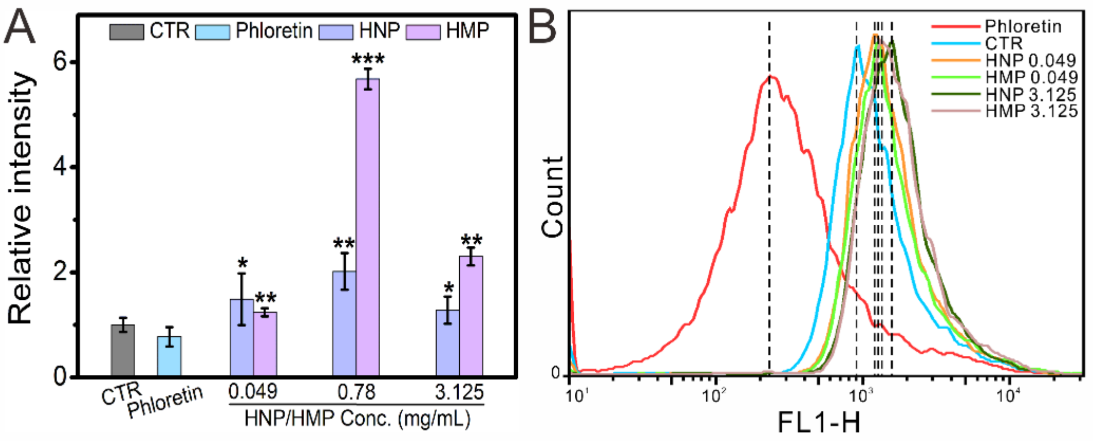

Figure S10. The glucose uptake quantification in chondrocytes. (A) Glucose uptake quantification by using image-pro plus software, (B) glucose uptake quantification by using flow cytometry. Repeat number: n = 6. In the experiment, the statistical analysis between CTR group and the other groups was conducted, *p < 0.05, **p < 0.01, ***p < 0.001 (one-way ANOVA followed by Dunnett's multiple comparisons test). Error bars represent mean±SD.

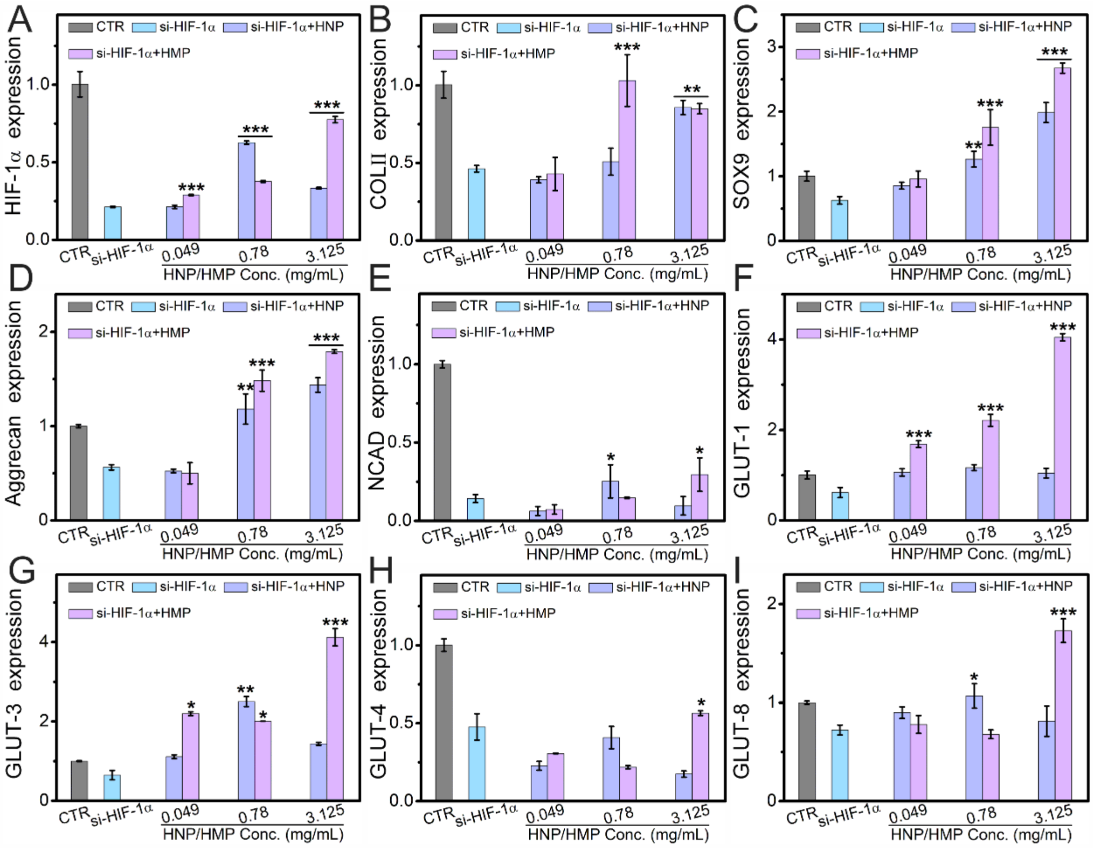

Figure S11. HNPs and HMPs stimulated HIF-1α and GLUT pathway related genes expressed in chondrocytes pretreating with si-HIF-1α. (A) HIF-1α, (B) COL II, (C) SOX9, (D) Aggrecan, (E) NCAD, (F) GLUT-1, (G) GLUT-3, (H) GLUT-4, (I) GLUT-8. As compared with si-HIF-1α group, the expression of HIF-1α in chondrocytes was significantly enhanced after co-cultured with HNPs/HMPs. Furthermore, the expression of cartilage specificity genes (COL II, SOX9, Aggrecan,

NCAD) and related genes (GLUT-1, GLUT-3, GLUT-4, GLUT-8) in GLUT pathway was obviously increased. Repeat number: n = 6. In the experiment, one-way ANOVA followed by Dunnett's multiple comparisons test (all groups against si-HIF-1α group) was used. (*$p < 0.05$, **$p < 0.01$, ***$p < 0.001$)

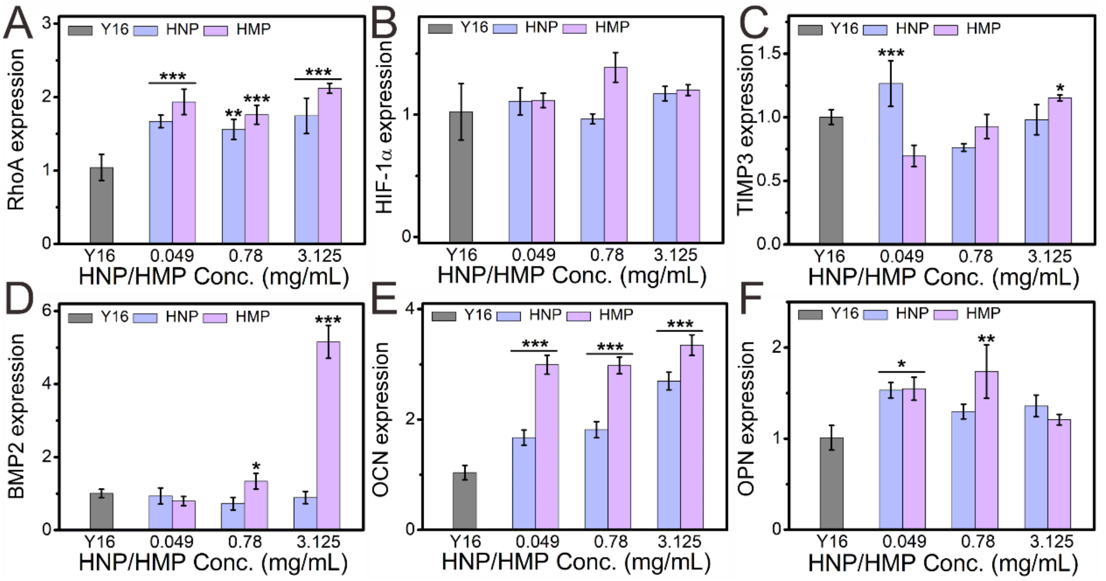

Figure S12. HNPs and HMPs enhanced RhoA expression in rBMSCs after pretreating with Y16. (A) RhoA, (B) HIF-1α, (C) TIMP3, (D) BMP2, (E) OCN, (F) OPN. As compared to pure Y16 treated group, HNP and HMP group significantly increased osteogenic differentiation by stimulating RhoA. Repeat number: n = 6. In the experiment, the statistical analysis between Y16 group and the other groups was conducted, *$p < 0.05$, **$p < 0.01$, ***$p < 0.001$ (one-way ANOVA followed by Dunnett's multiple comparisons test). Error bars represent mean±SD.

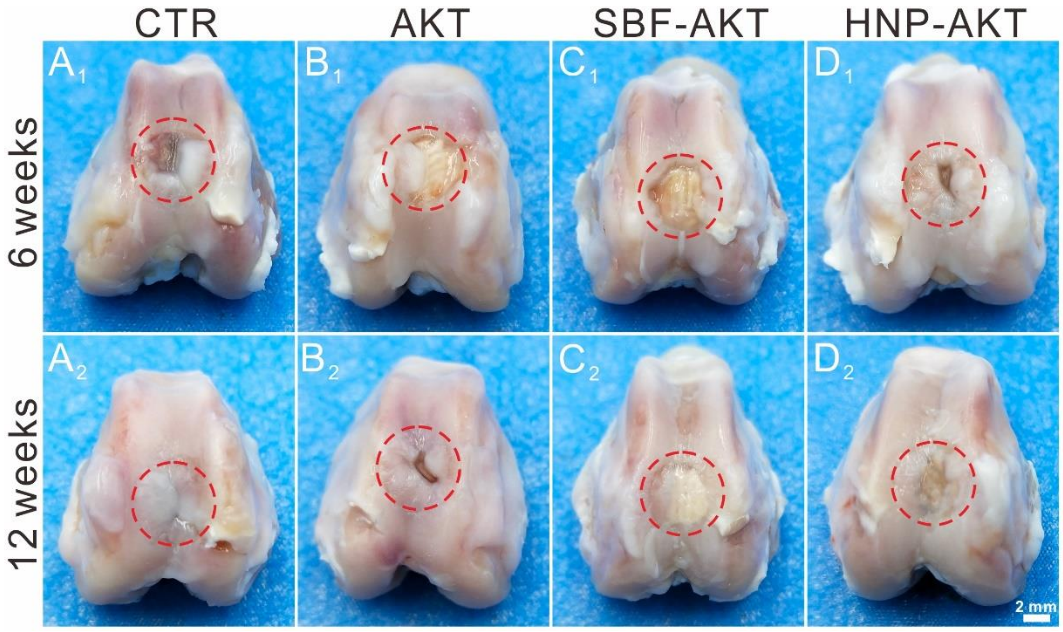

Figure S13. Photographs of the defect region. (A_{1-2}) CTR, (B_{1-2}) AKT group, (C_{1-2}) SBF-AKT

group, (D_{1-2}) HNP-AKT group.

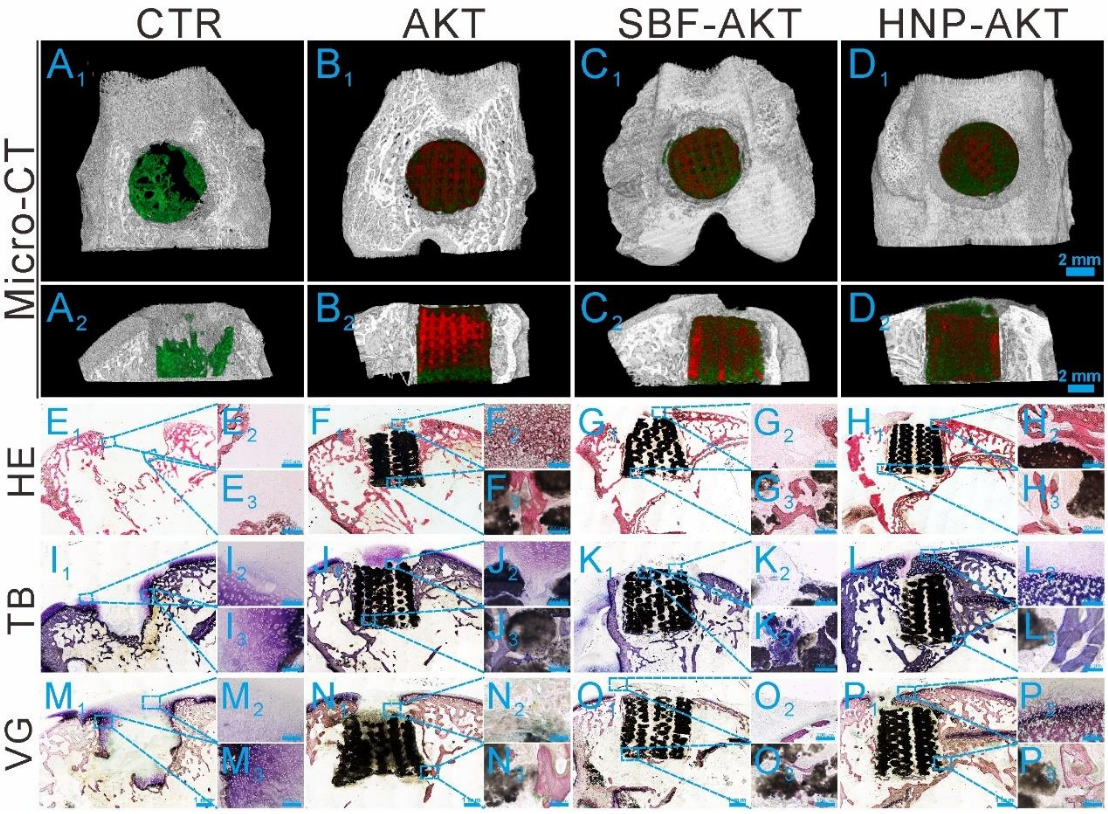

Figure S14. The osteochondral regeneration effect at 6 weeks. (A_1-D_2) showed the Micro-CT images of the defects. (A_1-D_1) and (A_2-D_2) showed the transverse view and sagittal view of Micro-CT images, respectively. Micro-CT images demonstrated that HNP-AKT group possessed much more calcified tissue than the other three experimental groups. (E_1-H_3) HE staining, (I_1-L_3) TB staining, (M_1-P_3) VG staining. The scale bar in Micro-CT image was 2 mm and the scale bars in the enlarge images of HE, TB and VG staining were 200 μm.

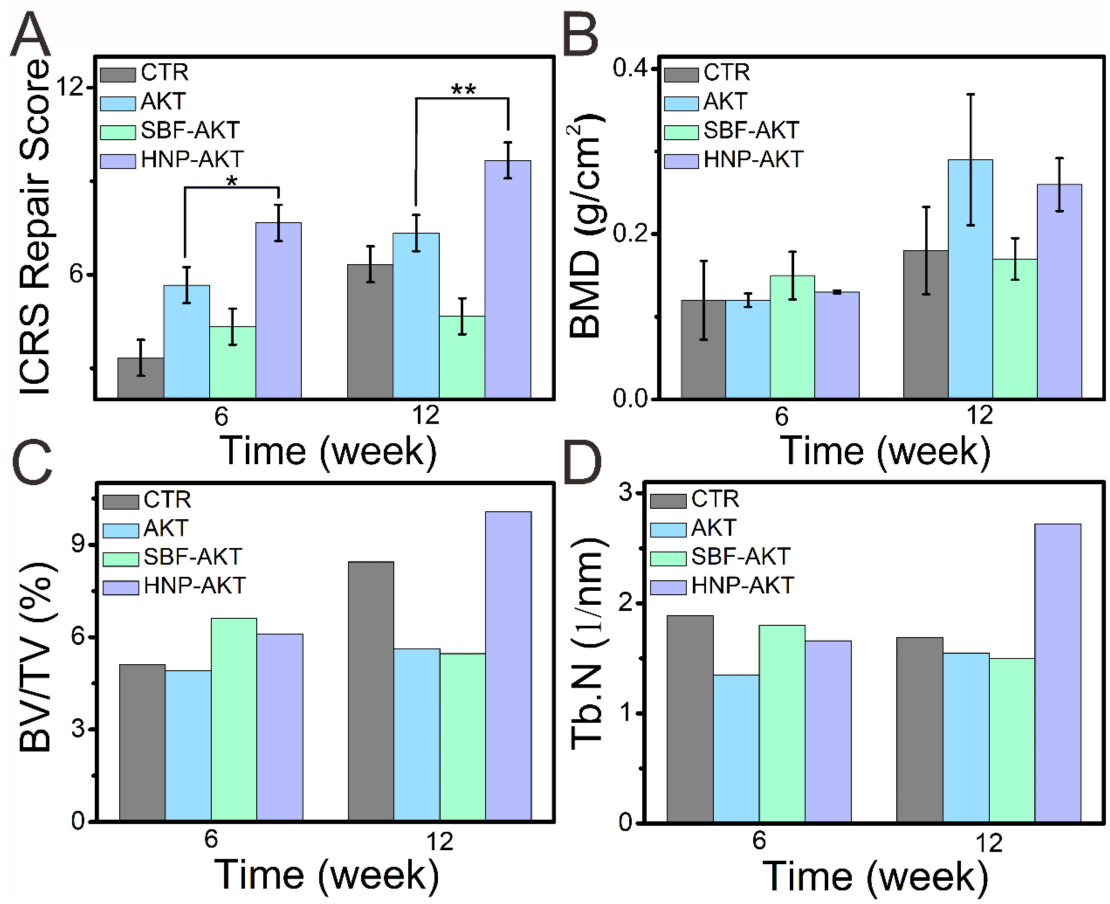

Figure S15. ICRS score and micro-CT analysis of the defects post-surgery. (A) ICRS score, (B) bone mineral density, (C) bone volume/total volume, (D) trabeculae number. ICRS score was blinded quantity by six investigators. Repeat number: n = 6. In the experiment, the statistical analysis between AKT group and the other groups was conducted, $*p < 0.05$, $**p < 0.01$ (one-way ANOVA followed by Dunnett's multiple comparisons test). Error bars represent mean±SD.

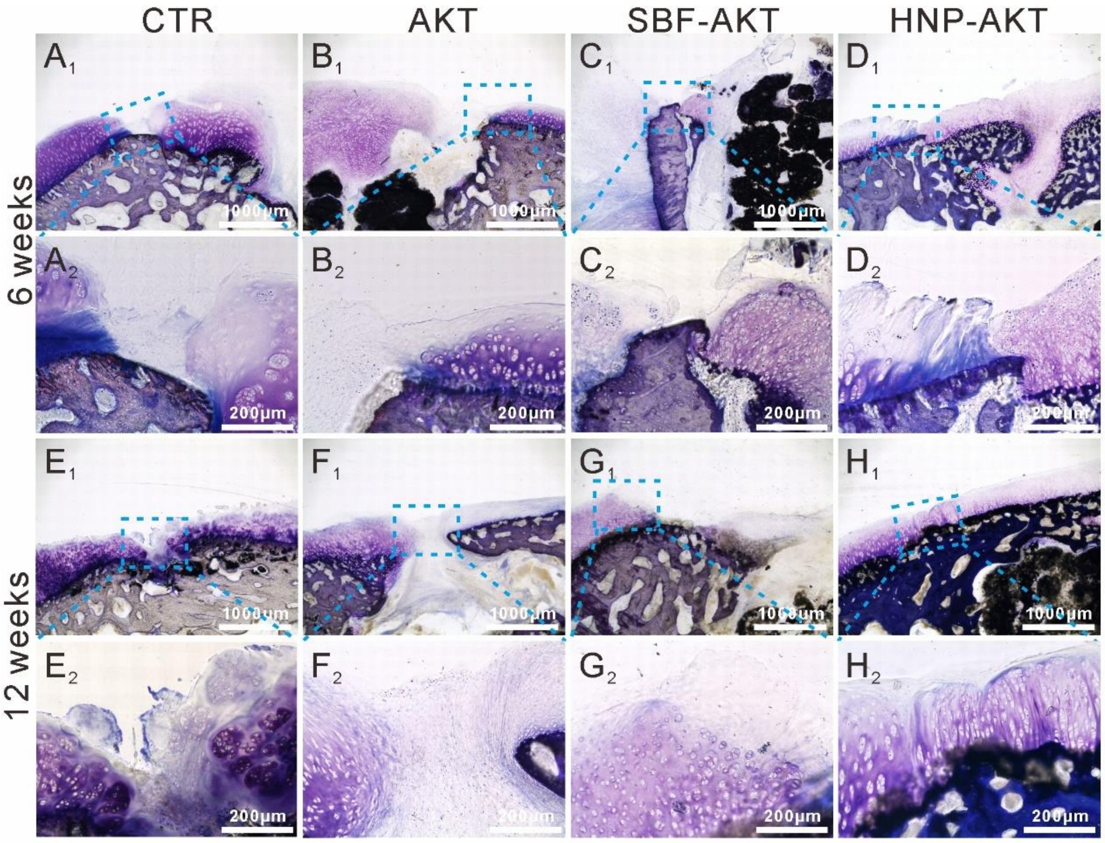

Figure S16. TB staining of the border zone in defects. (A_1-D_2) and (E_1-H_2) showed the TB staining of border zones after 6 and 12 weeks of repair, respectively. ($A_{1\text{-}2}$, $E_{1\text{-}2}$) CTR group, ($B_{1\text{-}2}$, $F_{1\text{-}2}$) AKT group, ($C_{1\text{-}2}$, $G_{1\text{-}2}$) SBF-AKT group, ($D_{1\text{-}2}$, $H_{1\text{-}2}$) HNP-AKT group. The purplish red color and blue color stand for cartilage and bone, respectively.

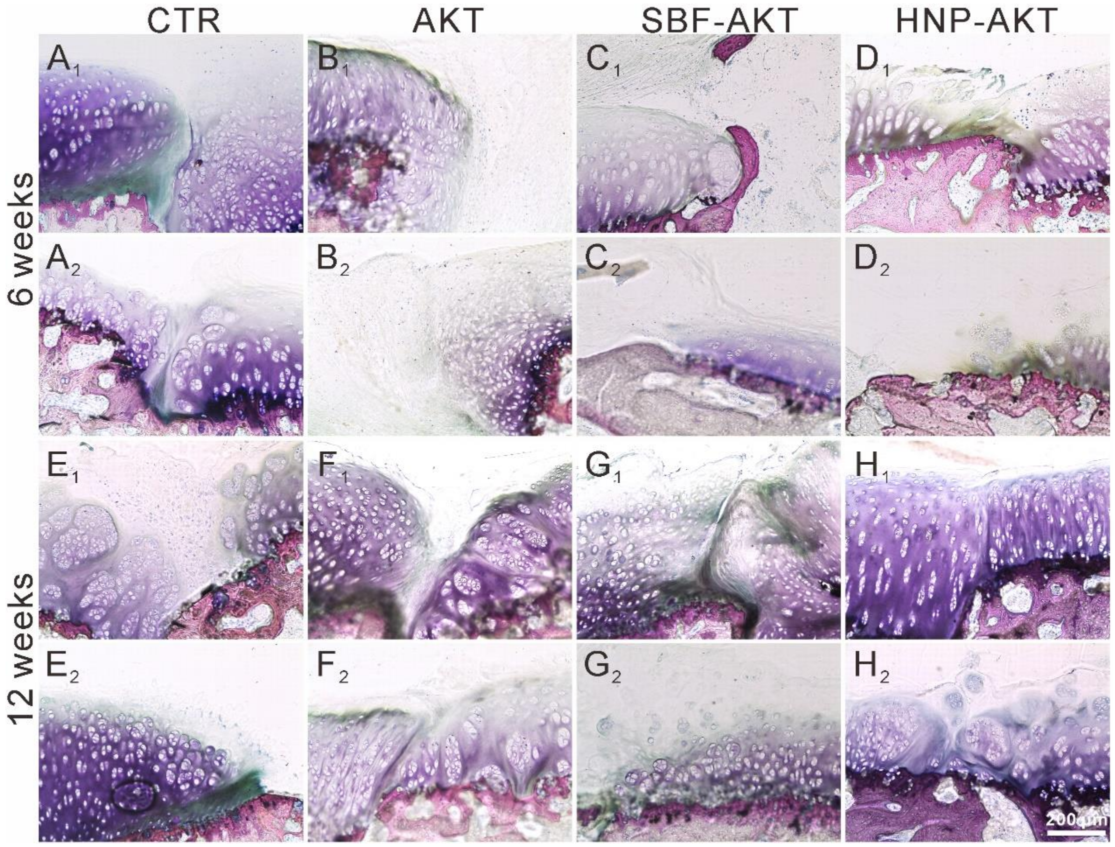

Figure S17. VG staining of the border zone in defects. (A_1-D_2) and (E_1-H_2) displayed the VG staining of border zones at week 6 and week 12 post-surgery, respectively. (A_{1-2}, E_{1-2}) CTR group, (B_{1-2}, F_{1-2}) AKT group, (C_{1-2}, G_{1-2}) SBF-AKT group, (D_{1-2}, H_{1-2}) HNP-AKT group. The green color, hyacinthine color and red color stand for collagen fiber, hyaline cartilage and bone tissue, respectively.
